# Predictive factors for alpha blocker use after transurethral prostatectomy: Can preoperative urodynamic outcome predict alpha blocker medication after surgery?

**DOI:** 10.1371/journal.pone.0274399

**Published:** 2022-09-21

**Authors:** Sung Jin Kim, Sung Gon Park, Sahyun Pak, Young Goo Lee, Sung Tae Cho, Ohseong Kwon

**Affiliations:** Department of Urology, Hallym University Kangnam Sacred Heart Hospital, Hallym University College of Medicine, Seoul, South Korea; Universidad Tecnológica de Pereira: Universidad Tecnologica de Pereira, COLOMBIA

## Abstract

**Objective:**

To analyze the diagnostic value of conducting urodynamic study (UDS) and show predictors for alpha blocker use 12 months after transurethral prostatectomy.

**Materials and methods:**

Our study includes 406 participants that had a transurethral prostatectomy at our hospital between 2010 and 2019. All participants took alpha blockers for more than a month. We collected the participants’ preoperative international prostatic symptom score (IPSS), uroflowmetry, transrectal ultrasound, and serum prostatic antigen (PSA) level. A total of 254 patients conducted UDS. After surgery, participants visited our hospital at 1,3,6, and 12 months.

**Results:**

133 patients (32.6%) took alpha blockers continuously for 12 months after surgery. They reported poor preoperative IPSS scores and uroflowmetry outcomes. They also had high postoperative PVR (40.68±24.56 vs 29.34±25.11, p<0.001) and total IPSS score (10.35±7.96 vs 8.43±6.74, p = 0.018) compared to the group which discontinued alpha blockers. A multivariate analysis (Table 2) found that conducting preoperative UDS (Odds ratio (OR) 6.067, p<0.001) Age>75 (OR 2.463, p<0.001), a history of taking 5-alpha reductase inhibitors (5-ARI) before surgery (OR 2.186 [95% CI 1.334–3.583], p = 0.002), IPSS item straining (OR 1.224, p = 0.003), duration of taking alpha blockers [OR 1.009, p = 0.020), and Qmax (OR 0.926, p = 0.018), PVR (OR 1.002, p = 0.022) were confirmed as a strong predictors of persistent alpha blocker use.

**Conclusion:**

Conducting preoperative UDS, Age>75, history of taking 5-ARI before surgery, IPSS item straining, duration of alpha blocker medication, Qmax, and PVR are possible determinant factors of alpha blocker use after surgery. By comparing UDS outcomes, detrusor underactivity can be a strong predictor of persisting alpha blocker therapy 12 months after surgery.

## Introduction

Alpha blockers are the most common drug for treating benign prostatic hyperplasia (BPH). They block stimulating alpha adrenergic receptors in the sympathetic nerve, which is known to block voiding by inducing contraction of the prostatic capsule and smooth muscle of the bladder neck [1, 2]. However, the effects of alpha blockers may vary in individuals depending on alpha-receptor density of the prostate and adenoma [3]. Some combination therapies are known to be effective in treating BPH. 5-alpha reductase inhibitor (5-ARI) is known to help improve symptoms when combined with alpha blockers [4, 5]. Antimuscarinics and beta-3 agonists are known to improve overactive bladder symptoms [6], and desmopressin reduces nocturia and nighttime frequency [5].

Surgical treatment can be considered for patients who are ineffective with drug treatment. Urodynamic study (UDS) is an invasive test that can help determine the cause of BPH [7]. UDS can diagnose bladder outlet obstruction (BOO) and determine the location of the blockages. When diagnosed with BOO, resolution of obstruction can improve urinary symptoms. We can also diagnose detrusor underactivity (DU) using UDS. DU is a known cause of poor efficacy after surgery, and the bladder contractility index can be used to diagnose DU. However, the relation between the outcome of UDS before surgery and the prognosis of surgery have not yet been clearly revealed.

About 20–50% of patients experience lower urinary tract symptoms (LUTS), even after TURP [8]. A number of patients continue to take BPH drugs for a period after surgery. This can have high social implications, directly affecting medical costs as well as personal daily functioning. This trend is more prominent in the elderly population [9]. Here, we analyze which preoperative factors are associated with continuing alpha blocker use in an elderly group, and if UDS can help predict the effects of alpha blocker medication after surgery.

## Materials and methods

This study was conducted with the IRB approval of our university hospital (HKS 2021-05-021-002). The present study was designed retrospectively between 2010 and 2019 on 406 patients diagnosed with BPH who complained of LUTS. Patients who underwent TURP before taking alpha blockers for at least a month were included in this study. We excluded patient those who diagnosed with prostate cancer and urethral stricture. We also excluded those who had previous prostatic surgery. Because the study was of a retrospective nature, informed consents were waived by the ethics committee, and all data were analyzed anonymously.

A total of 254 patients underwent UDS according to the standard method defined by the ICS [10]. The UDS exams were performed with a Duet® Logic G/2 device (Medtronic, Skovlunde, Denmark). Free uroflowmetric study was performed first after normal saline was aseptically infused into the bladder at a filling rate of 50 mL/min. We used normal saline at room temperature. A dual-lumen catheter of 6F was inserted (Medtronic Inc., Skovlunde, Denmark). To measure abdominal pressure, a rectal balloon catheter (Medical Measurement System) was inserted into the rectum at 10 cm above the anal verges. No vaginal catheter was used in women. A fixed unit with a Siemens-Arcadis 3D C-arm (Brainlab, AG, Munich, Germany) was used to obtain fluoroscopic images when necessary. One specialized urologist conducted all UDS procedures and interpreted results. According to the Schäfer nomogram, DU was defined using the bladder contractility index (BCI) with the following formula: BCI = 5Qmax + PdetQmax [11, 12]. DU was diagnosed if the BCI <100. BOO was defined as a BOOI score of >40 (BOOI = PdetQmax– 2Qmax) [11, 12].

We used the International Prostatic Symptom Score (IPSS) and uroflowmetry to evaluate preoperative subjective symptoms and objective measure, respectively. Transrectal ultrasound was conducted to measure prostatic size. Prostate size was measured transrectally using ellipsoid measurement on triplane axial. BVI-6100 (Verathon Inc, Bothell, WA) was used to measure bladder volume immediately after uroflowmetry. Residual urine volume value was generated from automatic calculation in cubic centimeters (cc).

Patients visited the outpatient clinic at 1, 3, 6, and 12 months after surgery if there were no specific issues. We performed uroflowmetry and IPSS when a patient complained of LUTS and required management. At 12months, uroflowmetry and IPSS were performed in all patients. Based on these results, we decided to discontinue medication. We defined patients who took alpha blockers “continuously” when alpha blockers were prescribed at every visit, without discontinuation, for up to 12 months. Group A is defined as the group who took alpha blockers continuously, and Group B are those who did not. Alpha blockers included tamsulosin, naftopidil, silodosin, alfuzosin, doxazosin and terazosin. We included antimuscarinics and beta-3 agonist as drugs to control OAB symptoms. We used following antimuscarinics in our study.: solifenacin, oxybutynin, tolterodine, fesoterodine, imidafenacin, and propiverin. We used only mirabegron as a beta-3 agonist in our study.

The Chi-square test was used to compare the patients’ previous drug history and rate of postoperative alpha blocker use between groups. The student-t test was used to compare the parameters of IPSS and uroflowmetry. Logistic regression analysis was performed on the preoperative items using the alpha blocker use history 12 months after surgery. Logistic regression analysis was performed on the preoperative items using the history of taking alpha blocker continuation. All statistical analysis was conducted using SPSS 27.0 (IBM Corp., Armonk, NY, USA).

## Results

Baseline characteristics are described in Table 1. There were no significant differences observed between the two groups in their underlying diseases, including neurological disease, hypertension, and diabetes. The age of Group A was slightly higher than that of Group B (75.47±7.95 vs 71.98±7.64, p<0.001). However, there was a significant difference in the duration of alpha blocker administration before surgery between Group A and Group B (29.67±43.22 vs 20.65±27.18, p = 0.029). The rate of 5-ARI use was lower in Group A (52 [39.1%] vs 152 [55.7%], p = 0.002), and the preoperative IPSS voiding symptom score (12.62±5.36 vs 11.13±5.58, p = 0.011) and storage symptom score (8.35±3.71 vs 7.54±3.87, p = 0.047) was significantly higher in Group A than in Group B. The preoperative Qmax (7.950±3.30 vs 9.52±4.74, p<0.001) and post-voided residual urine (PVR) (120.42±156.51 vs 88.48±99.57, p = 0.033) was significantly higher in Group A than Group B, while PSA level (2.90±3.03 vs 4.53±8.05, p = 0.003) and prostatic size (51.32±26.10 vs 57.95±29.25, p = 0.028) was lower in Group A. The conduction rate of UDS was more frequent in Group A (52 [39.1%] vs 202 [74.0%], p<0.001).

**Table 1:** Baseline characteristics.

		Total (n = 406)	Group A (n = 133)	Group B (n = 273)	p
Age		73.13±7.91	75.47±7.95	71.98±7.64	**<0.001**
BMI		24.40±3.07	24.00±3.14	24.59±3.01	0.071
ASA	1	83 (20.4%)	22 (16.5%)	61 (22.3%)	0.205
	2	272 (67.0%)	97 (72.9%)	175 (64.1%)	
	3,4	51 (12.6%)	14 (10.5%)	37 (13.6%)	
Cerebrovascular accident		54 (13.3%)	15 (11.3%)	39 (14.3%)	0.402
Hypertension		203 (50.0%)	65 (48.9%)	138 (50.5%)	0.751
Diabetes mellitus		93 (22.9%)	33 (24.8%)	60 (22.0%)	0.524
Spinal degenerative disease		48 (11.8%)	20 (15.0%)	28 (10.3%)	0.161
Previous AUR		37 (8.9%)	12 (9.0%)	24 (8.8%)	0.939
*Preoperative medication*					
Duration of taking alpha blocker		23.60±33.51	29.67±43.22	20.65±27.18	**0.029**
Alpha blocker monotherapy		140 (34.4%)	53 (39.8%)	87 (31.9%)	0.112
Combination therapy					
5-ARI		204 (50.2%)	52 (39.1%)	152 (55.7%)	**0.002**
Drugs to control OAB symptoms		104 (25.6%)	34 (28.6%)	66 (24.2%)	0.341
Desmopressin		14 (3.4%)	4 (3.0%)	10 (3.7%)	0.734
*Subjective measures*					
IPSS 3 (Intermittency)		2.81±1.79	3.08±1.69	2.68±1.82	**0.034**
IPSS 5 (Weak stream)		3.60±1.63	3.94±1.39	3.43±1.70	**0.001**
IPSS 6 (Straining)		2.50±1.84	2.86±1.78	2.33±1.85	**0.006**
IPSS 7 (Nocturia)		2.65±1.39	2.96±1.34	2.49±1.39	**0.001**
IPSS QoL		4.00±1.09	4.02±1.17	3.98±1.05	0.724
Voiding symptom score		11.62±5.55	12.62±5.36	11.13±5.58	**0.011**
Storage symptom score		7.81±3.84	8.35±3.71	7.54±3.87	**0.047**
Total score		19.42±8.21	20.96±8.05	18.67±8.20	**0.008**
*Objective measures*					
Uroflowmetry	Qmax	9.01±4.38	7.950±3.30	9.52±4.74	**<0.001**
	PVR	98.945±121.93	120.42±156.51	88.48±99.57	**0.033**
PSA		4.00±6.86	2.90±3.03	4.53±8.05	**0.003**
Prostate size		55.789±28.40	51.32±26.10	57.95±29.25	**0.028**
Conducting preoperative UDS		254 (62.6%)	52 (39.1%)	202 (74.0%)	**<0.001**

Abbreviations: BMI, body mass index; ASA, American society of anesthesiology score; AUR, history of acute urinary retention; 5-ARI, 5-alpha reductase inhibitor; OAB, overactive bladder; IPSS, international prostatic symptom score; PSA, prostatic serum antigen; Qmax, maximum urine rate; PVR, postvoided-residual urine; UDS, urodynamic study

The multivariate analysis shown in Table 2. It shows that preoperative UDS conduction (Odds ratio (OR) 6.067 [95% confidence interval(CI) 3.613–10.188], p<0.001), Age>75 (OR 2.463 [95% CI 1.490–4.073], p<0.001), history of taking 5-ARI before surgery (OR 2.186 [95% CI 1.334–3.583], p = 0.002), IPSS item straining (OR 1.224 [95% CI 1.070–1.401], p = 0.003), duration of alpha blocker use [OR 1.009 (95% CI 1.001–1.017], p = 0.020), Qmax [OR 0.926 [95% CI 0.0.869–0.987]. p = 0.018), and PVR (OR 1.002 [95% CI 1.000–1.004], p = 0.022) were confirmed as strong predictors for persistent use of alpha blockers.

**Table 2:** Logistic regression analysis examining the relationship between baseline characteristics and taking alpha blockers continuously at 12 months postoperatively.

	univariate analysis					multivariate analysis				
	B	p	Odds	95% CI		B	p	Odds	95% CI	
Age >75	0.789	**<0.001**	2.201	1.443	3.355	0.902	**<0.001**	2.463	1.490	4.073
duration of taking alpha blocker	0.008	**0.014**	1.008	1.002	1.014	0.009	**0.020**	1.009	1.001	1.017
History of preoperative 5-ARI	0.671	**0.002**	1.957	1.283	2.985	0.782	**0.002**	2.186	1.334	3.583
IPSS Intermittency	0.128	**0.034**	1.136	1.009	1.279					
IPSS Weak stream	0.207	**0.003**	1.229	1.070	1.412					
IPSS Straining	0.158	**0.007**	1.172	1.044	1.314	0.202	**0.003**	1.224	1.070	1.401
IPSS Nocturia	0.247	**0.002**	1.281	1.099	1.493					
PSA	-0.063	**0.025**	0.939	0.888	0.992					
Qmax	-0.094	**0.001**	0.910	0.862	0.962	-0.077	**0.018**	0.926	0.869	0.987
PVR	0.002	**0.017**	1.002	1.000	1.004	0.002	**0.022**	1.002	1.000	1.004
Prostatic size	-0.009	**0.030**	0.991	0.983	0.999					
Conducting preoperative UDS	1.489	**<0.001**	4.432	2.852	6.888	1.803	**<0.001**	6.067	3.613	10.188

Abbreviations: 5-ARI Hx, history of preoperative 5-alpha reductase inhibitor; IPSS, international prostatic symptom score; PSA, prostatic serum antigen; Qmax, maximum urine rate; PVR, postvoided-residual urine; UDS, urodynamic study

Among the 254 patients who underwent UDS before surgery, Group A had less BOO diagnoses (13/52 [9.8%] vs 82/202 [30.0%], p = 0.038) and a higher rate of DU diagnoses (45/52 [86.5%] vs 134/202 [66.3%], p = 0.004) compared with Group B (n = 202). By comparing UDS outcomes, patients were classified into only diagnosed BOO, only diagnosed DU, and those diagnosed with BOO and DU (BOO+DU). There was a significant difference in taking alpha blockers from 3 months after surgery in the 3 groups, as shown in Table 3.

**Table 3:** Comparison between rate of alpha blocker use postoperatively depending on urodynamic outcomes.

Postoperative	non-UDS (n = 152)	UDS (n = 254)					p
		BOO (n = 44)	DU (n = 128)	BOO+DU (n = 51)	total	p	
1month	125 (82.2%)	24 (54.5%)	85 (66.4%)	31 (60.8%)	156 (61.4%)	0.322	**<0.001**
3months	124 (81.6%)	14 (31.8%)	58 (45.3%)	22 (43.1%)	100 (39.4%)	**0.038**	**<0.001**
6months	102 (67.1%)	8 (18.2%)	46 (35.9%)	15 (29.4%)	73 (28.7%)	**0.024**	**<0.001**
12months	98 (64.5%)	6 (13.6%)	54 (42.2%)	16 (31.4%)	81 (31.9%)	**0.001**	**<0.001**
Taking alpha blocker until 12months postoperatively	81 (53.3%)	3 (6.8%)	35 (27.3%)	10 (19.6%)	52 (20.5%)	**0.020**	**<0.001**

Abbreviations: UDS, urodynamic study; BOO, bladder outlet obstruction; DU, detrusor underactivity; BOO+DU, bladder outlet obstruction with detrusor underactivity; non-UDS: the group that didn’t undergo UDS, UDS: the group that underwent UDS

In Group A, PVR was found to be higher (40.68±24.56 vs 29.34±25.11, p<0.001) and Qmax was lower (15.98±7.51 vs 18.48±8.75, p = 0.003) than Group B at 12 months postoperatively. The total IPSS score of Group A (10.35±7.96 vs 8.43±6.74, p = 0.018) was significantly higher than that of Group B, as shown in Table 4.

**Table 4:** Postoperative outcomes at 12months postoperatively.

	Group A (n = 133)	Group B (n = 273)	p
IPSS voiding symptom score	5.55±5.37	4.19±4.38	**0.012**
IPSS storage symptom score	4.80±3.34	4.24±3.34	0.101
IPSS Total score	10.35±7.96	8.43±6.74	**0.018**
△IPSS Total score	-10.62±8.21	-10.24±8.28	0.665
IPSS QoL score	2.48±1.34	2.50±1.34	0.882
IPSS △QoL score	-1.89±1.88	-1.75±1.46	0.425
Qmax	15.98±7.51	18.48±8.75	**0.003**
△Qmax	8.03±7.94	8.96±8.02	0.270
PVR	40.68±24.56	29.34±25.11	**<0.001**
△PVR	-79.74±155.78	-59.14±102.31	0.167

Abbreviations: IPSS, international prostatic symptom score; QoL, IPSS item quality of life; Qmax, maximum urine flow rate; PVR, postvoided-residual urine

## Discussion

Patients who complained of LUTS expect surgery to allow them to discontinue the drug. However, it is known that a large number of patients still took the drug one year after operation, and the rate of drug use gradually increases as time goes by [13]. Alpha blockers are the most commonly used drugs after TURP according to many studies [8, 13, 14]. However, they have no impact on outlet obstruction progression, and their efficacy usually decreases [15]. In addition, continuous use of alpha blockers may cause dementia, depression, and sexual dysfunction [16, 17]. We designed our study to find predicting factors for continuous alpha blocker use after transurethral prostatectomy and to determine the characteristics of sustained alpha blocker use.

Interestingly, our study found that history of conducting preoperative UDS can significantly associated with postoperative alpha blocker use (Tables 1 and 2). In the current guidelines, UDS is not an initially recommend diagnostic tool to evaluate BPH after a complaint of LUTS [7]. However, UDS can be a great diagnostic tool to measure the voiding condition before surgery. We didn’t find any reports that linked UDS before surgery to a good prognosis after surgery. In the Cochrane study, 350 patients were enrolled to determine if preoperative UDS was useful in BPH surgery [18]. The group that received only clinical evaluation had a significantly higher probability of occlusion 6 months after surgery than the group that additionally conducted UDS before surgery (relative risk 0.87, 95% CI 0.83–0.92); however, there was missing data in this study (missing for 24 of 188 men in one arm). In UPSTREAM trial, IPSS score, Qmax at 18 months after surgery and the number of operations performed were similar between the UDS group and routine-care group [19]. It is unclear whether preoperative UDS predicted possible postoperative complications or symptoms after treatment. Our study is meaningful in suggesting that conducting UDS may reduce the use of alpha blockers: one of the most common prescriptions after surgery.

When we compare outcomes of UDS, patients diagnosed with DU (DU, DU+BOO) are more commonly prescribed postoperative alpha blockers than patients with BOO alone (Table 3). This implies that DU can cause postoperative use of alpha blockers. There is a meta-analysis that analyzed the efficacy after undergoing TURP in those diagnosed with DU preoperatively [20]. It shows that DU patients had worse Qmax and IPSS changes than non DU patients. However, it also showed that postoperative uroflowmetry and IPSS were improved compared to preoperative measures. This verifies that it is helpful to proceed with surgery, even in DU patients who were not diagnosed with BOO.

Alpha blockers used postoperatively effects alpha-adrenergic receptors and denervates the residual prostate and bladder neck involving smooth muscle relaxation, even if the prostate is surgically resected. Alpha blockers are also known to affect the spinal cord, ganglia, and nerve terminals [21]. Although alpha blockers are not considered standard drugs for DU, few animal experiments and a clinical study show that alpha blockers may improve postoperative outcomes in patients with DU. Alpha blockers improved voiding function in two types of male rats that induced DU [22, 23]. In a clinical study, the postoperative IPSS score in a group taking alpha blockers was significantly better than the baseline score in DU male patients (changed from 12.33±6.01 for pretreatment to 8.67±6.26 for post-treatment, p = 0.0009) [24]. However, the Qmax (changed from 3.60±2.10 for pretreatment to 3.59±1.96 for post-treatment, p = 0.9707) and PVR (changed from 191.70±155.20 for pretreatment to 159.80±134.80 for post-treatment, p = 0.0838) did not differ before and after treatment, likely because patients with BOO were excluded from the analyses.

High PVR is not always related to BOO, but may be related to DU. (7) Alpha blockers are believed to reduce elevated PVR induced by DU. Han et al, showed that the group taking the BPH drug for more than 3 months had a higher PVR after surgery than the group who had previously stopped taking the drug before 3 months (46.6±67.7 for medication group vs 22.1±32.1 for medication-free group, p<0.001) [25]. This study suggests that there may be a relationship between DU in the group persistently taking the BPH drug, but it does not specifically suggest a relationship between taking alpha blockers and DU.

In our study, we included patients who failed combination therapy with alpha blockers and 5-ARI. A total 204 patients (50.2%) received combination therapy with 5-ARI prior to surgery. 5-ARI works selectively to inhibit the 5-alpha reductase isoenzyme to reduce dihydrotestosterone. A change of hormones causes a decrease in the size of the prostate. Combination therapy with 5-ARI is known to relieve storage symptoms and reduce the frequency of AUR in the preoperative setting [26]. Taking 5-ARI is also known to lower some postoperative complications under special circumstances. 5-ARI acts on the prostate to reduce microvascular density and decrease blood flow to the prostate [26]. Preoperative use of finasteride is believed to reduce perioperative blood loss, but the effectiveness of dutasteride is controversial [27, 28]. Welk et al show that 5-ARI does not reduce postoperative gross hematuria [29]. Preoperative 5-ARI administration is expected to reduce prostate size and postoperative complications.

In our study, the total IPSS was higher in the group taking alpha blockers continuously after surgery than the group who did not. When comparing each item of IPSS, we found that the voiding symptom score was higher in the alpha blocker group. We believe that alpha blockers were taken to resolve these symptoms. The cause of voiding LUTS immediately after surgery may be a urinary tract infection or urethral stricture [30]. However, if symptoms persist after sufficient time has elapsed since surgery, invasive tests, such as transrectal ultrasound or UDS, may be required to determine the cause of LUTS as well as cystourethroscopy.

Our study has some limitations. First, this study is retrospective. Second, we used BCI, the most common method to define DU, to classify urodynamic outcomes. However, consensus on the definition of DU has not yet been conclusive. Our research results may sufficiently change depending on the results of future consensus. Third, we prescribed alpha blockers based on the symptoms at the follow-up day, but subjective factors, such as patient preference, may not be reflected. Objective measures that can exclude the patient’s subjective factors were not reflected in drug prescription.

In conclusion, conducting preoperative UDS, those aged more than 75-years, have a history of taking 5-ARI before surgery, IPSS item straining, duration of alpha blocker medication, Qmax, and PVR are possible determinant factors of alpha blocker use after surgery. When comparing the group that underwent UDS, the alpha blocker group had a higher rate of DU. Assuming that PVR was increased in the alpha blocker group, DU can be a strong predictor of persisting alpha blocker therapy continuously for 12 months after surgery.

## Supporting information

S1 Table Statistic information for Table 1. (DOCX)

S2 Table Statistic information for Table 2. (DOCX)

S3 Table Statistic information for Table 3. (DOCX)

S4 Table Statistic information for Table 4. (DOCX)

## Abbreviations

UDS: urodynamic study
TURP: transurethral resection of prostate
PSA: prostatic specific antigen
IPSS: international prostatic symptom score
OR: odds ratio
BPH: benign prostatic hyperplasia
LUTS: lower urinary tract symptom
BCI: bladder contractility index
DU: detrusor underactivity
BOOI: bladder outlet obstruction index
BOO: bladder outlet obstruction
Qmax: Maximum urine flow rate
PVR: post-voided residual urine
5-ARI: 5-alpha reductase inhibitor
