# Supplementary material for: Predictive factors for alpha blocker use after transurethral prostatectomy: Can preoperative urodynamic outcome predict alpha blocker medication after surgery?
Source: PLoS One. 2022 Sep 21;17(9):e0274399. doi: 10.1371/journal.pone.0274399 (PMC9491595; doi:10.1371/journal.pone.0274399)
Supplement: S1 Table — (DOCX) [file pone.0274399.s001.docx]

| **Descriptive Statistics** | | | |
| --- | --- | --- | --- |
|  | N | Mean | Std. Deviation |
| Age | 406 | 73.13 | 7.908 |
| BMI | 406 | 24.395304973243004 | 3.064869071463046 |
| Duration of taking alpha blocker | 406 | 23.60 | 33.506 |
| Pre IPSS Q3 | 406 | 2.81 | 1.789 |
| Pre IPSS Q5 | 406 | 3.60 | 1.625 |
| Pre IPSS Q6 | 406 | 2.50 | 1.838 |
| Pre IPSS Q7 | 406 | 2.65 | 1.387 |
| Pre IPSS Q8 | 406 | 4.00 | 1.091 |
| IPSS VS | 406 | 11.62 | 5.545 |
| IPSS SS | 406 | 7.81 | 3.835 |
| IPSS total | 406 | 19.42 | 8.210 |
| Qmax | 406 | 9.008 | 4.3804 |
| PVR | 406 | 98.945 | 121.9291 |
| PSA | 406 | 4.00 | 6.861 |
| Prostate size | 405 | 55.7867 | 28.40325 |
| Valid N (listwise) | 405 |  |  |

T-Test

| **Notes** | | |
| --- | --- | --- |
| Output Created | | 07-JUL-2022 17:08:19 |
| Comments | |  |
| Input | Data | C:\Users\JohnS\Desktop\alpha blocker UDS final raw.sav |
|  | Active Dataset | 데이터세트1 |
|  | Filter | <none> |
|  | Weight | <none> |
|  | Split File | <none> |
|  | N of Rows in Working Data File | 406 |
| Missing Value Handling | Definition of Missing | User defined missing values are treated as missing. |
|  | Cases Used | Statistics for each analysis are based on the cases with no missing or out-of-range data for any variable in the analysis. |
| Syntax | | T-TEST GROUPS=Alphablockercontinuationtil1yr(0 1)  /MISSING=ANALYSIS  /VARIABLES=age BMI PreIPSSQ3 PreIPSSQ5 PreIPSSQ6 PreIPSSQ7 PreIPSSQ8 IPSSVS IPSSSS IPSStotal Qmax  PVR PSA Prostatesize  /ES DISPLAY(TRUE)  /CRITERIA=CI(.95). |
| Resources | Processor Time | 00:00:00.02 |
|  | Elapsed Time | 00:00:00.02 |

| **Group Statistics** | | | | | |
| --- | --- | --- | --- | --- | --- |
|  | Alpha blocker continuation til 1yr | N | Mean | Std. Deviation | Std. Error Mean |
| age | 0 | 273 | 71.98 | 7.642 | .463 |
|  | 1 | 133 | 75.47 | 7.954 | .690 |
| BMI | 0 | 273 | 24.586742298243024 | 3.013027079570022 | .182356694523245 |
|  | 1 | 133 | 24.002354674558653 | 3.143642255104586 | .272588354001847 |
| Pre IPSS Q3 | 0 | 273 | 2.68 | 1.822 | .110 |
|  | 1 | 133 | 3.08 | 1.692 | .147 |
| Pre IPSS Q5 | 0 | 273 | 3.43 | 1.707 | .103 |
|  | 1 | 133 | 3.94 | 1.386 | .120 |
| Pre IPSS Q6 | 0 | 273 | 2.33 | 1.845 | .112 |
|  | 1 | 133 | 2.86 | 1.776 | .154 |
| Pre IPSS Q7 | 0 | 273 | 2.49 | 1.386 | .084 |
|  | 1 | 133 | 2.96 | 1.339 | .116 |
| Pre IPSS Q8 | 0 | 273 | 3.98 | 1.052 | .064 |
|  | 1 | 133 | 4.02 | 1.171 | .102 |
| IPSS VS | 0 | 273 | 11.13 | 5.580 | .338 |
|  | 1 | 133 | 12.62 | 5.357 | .464 |
| IPSS SS | 0 | 273 | 7.54 | 3.873 | .234 |
|  | 1 | 133 | 8.35 | 3.713 | .322 |
| IPSS total | 0 | 273 | 18.67 | 8.195 | .496 |
|  | 1 | 133 | 20.96 | 8.054 | .698 |
| Qmax | 0 | 273 | 9.523 | 4.7404 | .2869 |
|  | 1 | 133 | 7.950 | 3.3001 | .2862 |
| PVR | 0 | 273 | 88.482 | 99.5672 | 6.0261 |
|  | 1 | 133 | 120.421 | 156.5073 | 13.5709 |
| PSA | 0 | 273 | 4.53 | 8.047 | .487 |
|  | 1 | 133 | 2.90 | 3.032 | .263 |
| Prostate size | 0 | 273 | 57.9471 | 29.25287 | 1.77046 |
|  | 1 | 132 | 51.3184 | 26.10262 | 2.27194 |

| **Independent Samples Test** | | | | | | | | | | |
| --- | --- | --- | --- | --- | --- | --- | --- | --- | --- | --- |
|  | | Levene's Test for Equality of Variances | | t-test for Equality of Means | | | | | | |
|  |  | F | Sig. | t | df | Sig. (2-tailed) | Mean Difference | Std. Error Difference | 95% Confidence Interval of the Difference | |
|  |  |  |  |  |  |  |  |  | Lower | Upper |
| age | Equal variances assumed | .124 | .725 | -4.263 | 404 | .000 | -3.492 | .819 | -5.102 | -1.882 |
|  | Equal variances not assumed |  |  | -4.205 | 252.639 | .000 | -3.492 | .830 | -5.127 | -1.857 |
| BMI | Equal variances assumed | .377 | .540 | 1.808 | 404 | .071 | .584387623684371 | .323187324228768 | -.050951236049608 | 1.219726483418351 |
|  | Equal variances not assumed |  |  | 1.782 | 252.085 | .076 | .584387623684371 | .327960934830477 | -.061504920279873 | 1.230280167648616 |
| Pre IPSS Q3 | Equal variances assumed | 2.562 | .110 | -2.131 | 404 | .034 | -.401 | .188 | -.772 | -.031 |
|  | Equal variances not assumed |  |  | -2.186 | 279.893 | .030 | -.401 | .184 | -.763 | -.040 |
| Pre IPSS Q5 | Equal variances assumed | 17.232 | .000 | -2.983 | 404 | .003 | -.508 | .170 | -.842 | -.173 |
|  | Equal variances not assumed |  |  | -3.203 | 315.650 | .001 | -.508 | .158 | -.819 | -.196 |
| Pre IPSS Q6 | Equal variances assumed | .538 | .464 | -2.736 | 404 | .006 | -.527 | .193 | -.906 | -.149 |
|  | Equal variances not assumed |  |  | -2.773 | 271.022 | .006 | -.527 | .190 | -.902 | -.153 |
| Pre IPSS Q7 | Equal variances assumed | 1.033 | .310 | -3.228 | 404 | .001 | -.468 | .145 | -.753 | -.183 |
|  | Equal variances not assumed |  |  | -3.266 | 269.921 | .001 | -.468 | .143 | -.750 | -.186 |
| Pre IPSS Q8 | Equal variances assumed | 3.768 | .053 | -.354 | 404 | .724 | -.041 | .115 | -.268 | .186 |
|  | Equal variances not assumed |  |  | -.341 | 238.331 | .733 | -.041 | .120 | -.277 | .195 |
| IPSS VS | Equal variances assumed | .509 | .476 | -2.555 | 404 | .011 | -1.488 | .582 | -2.633 | -.343 |
|  | Equal variances not assumed |  |  | -2.592 | 271.612 | .010 | -1.488 | .574 | -2.619 | -.358 |
| IPSS SS | Equal variances assumed | .702 | .403 | -1.989 | 404 | .047 | -.804 | .404 | -1.598 | -.009 |
|  | Equal variances not assumed |  |  | -2.018 | 271.918 | .045 | -.804 | .398 | -1.588 | -.020 |
| IPSS total | Equal variances assumed | .043 | .836 | -2.660 | 404 | .008 | -2.292 | .862 | -3.986 | -.598 |
|  | Equal variances not assumed |  |  | -2.676 | 265.900 | .008 | -2.292 | .857 | -3.979 | -.606 |
| Qmax | Equal variances assumed | 8.172 | .004 | 3.441 | 404 | .001 | 1.5731 | .4571 | .6744 | 2.4717 |
|  | Equal variances not assumed |  |  | 3.882 | 356.127 | .000 | 1.5731 | .4052 | .7762 | 2.3700 |
| PVR | Equal variances assumed | 16.316 | .000 | -2.493 | 404 | .013 | -31.9390 | 12.8110 | -57.1236 | -6.7544 |
|  | Equal variances not assumed |  |  | -2.151 | 185.683 | .033 | -31.9390 | 14.8487 | -61.2328 | -2.6452 |
| PSA | Equal variances assumed | 7.974 | .005 | 2.253 | 404 | .025 | 1.626 | .722 | .207 | 3.045 |
|  | Equal variances not assumed |  |  | 2.938 | 386.088 | .003 | 1.626 | .553 | .538 | 2.714 |
| Prostate size | Equal variances assumed | 1.713 | .191 | 2.212 | 403 | .028 | 6.62873 | 2.99671 | .73760 | 12.51987 |
|  | Equal variances not assumed |  |  | 2.301 | 287.374 | .022 | 6.62873 | 2.88032 | .95953 | 12.29794 |

| **Independent Samples Effect Sizes** | | | | | |
| --- | --- | --- | --- | --- | --- |
|  | | Standardizer^a^ | Point Estimate | 95% Confidence Interval | |
|  |  |  |  | Lower | Upper |
| age | Cohen's d | 7.746 | -.451 | -.660 | -.241 |
|  | Hedges' correction | 7.760 | -.450 | -.659 | -.241 |
|  | Glass's delta | 7.954 | -.439 | -.652 | -.224 |
| BMI | Cohen's d | 3.056317345112717 | .191 | -.017 | .399 |
|  | Hedges' correction | 3.062005869059156 | .191 | -.017 | .398 |
|  | Glass's delta | 3.143642255104586 | .186 | -.023 | .394 |
| Pre IPSS Q3 | Cohen's d | 1.781 | -.225 | -.433 | -.017 |
|  | Hedges' correction | 1.784 | -.225 | -.432 | -.017 |
|  | Glass's delta | 1.692 | -.237 | -.446 | -.028 |
| Pre IPSS Q5 | Cohen's d | 1.609 | -.315 | -.524 | -.107 |
|  | Hedges' correction | 1.612 | -.315 | -.523 | -.107 |
|  | Glass's delta | 1.386 | -.366 | -.578 | -.154 |
| Pre IPSS Q6 | Cohen's d | 1.823 | -.289 | -.497 | -.081 |
|  | Hedges' correction | 1.826 | -.289 | -.496 | -.081 |
|  | Glass's delta | 1.776 | -.297 | -.507 | -.086 |
| Pre IPSS Q7 | Cohen's d | 1.371 | -.341 | -.550 | -.133 |
|  | Hedges' correction | 1.373 | -.341 | -.549 | -.132 |
|  | Glass's delta | 1.339 | -.349 | -.560 | -.137 |
| Pre IPSS Q8 | Cohen's d | 1.092 | -.037 | -.245 | .170 |
|  | Hedges' correction | 1.094 | -.037 | -.244 | .170 |
|  | Glass's delta | 1.171 | -.035 | -.242 | .172 |
| IPSS VS | Cohen's d | 5.508 | -.270 | -.478 | -.062 |
|  | Hedges' correction | 5.518 | -.270 | -.477 | -.062 |
|  | Glass's delta | 5.357 | -.278 | -.487 | -.067 |
| IPSS SS | Cohen's d | 3.821 | -.210 | -.418 | -.002 |
|  | Hedges' correction | 3.829 | -.210 | -.417 | -.002 |
|  | Glass's delta | 3.713 | -.216 | -.425 | -.007 |
| IPSS total | Cohen's d | 8.149 | -.281 | -.489 | -.073 |
|  | Hedges' correction | 8.164 | -.281 | -.488 | -.073 |
|  | Glass's delta | 8.054 | -.285 | -.494 | -.074 |
| Qmax | Cohen's d | 4.3229 | .364 | .155 | .572 |
|  | Hedges' correction | 4.3309 | .363 | .155 | .571 |
|  | Glass's delta | 3.3001 | .477 | .261 | .691 |
| PVR | Cohen's d | 121.1515 | -.264 | -.472 | -.055 |
|  | Hedges' correction | 121.3770 | -.263 | -.471 | -.055 |
|  | Glass's delta | 156.5073 | -.204 | -.412 | .005 |
| PSA | Cohen's d | 6.826 | .238 | .030 | .446 |
|  | Hedges' correction | 6.839 | .238 | .030 | .445 |
|  | Glass's delta | 3.032 | .536 | .318 | .752 |
| Prostate size | Cohen's d | 28.26738 | .235 | .026 | .443 |
|  | Hedges' correction | 28.32013 | .234 | .026 | .442 |
|  | Glass's delta | 26.10262 | .254 | .043 | .464 |
| a. The denominator used in estimating the effect sizes.  Cohen's d uses the pooled standard deviation.  Hedges' correction uses the pooled standard deviation, plus a correction factor.  Glass's delta uses the sample standard deviation of the control group. | | | | | |

CROSSTABS

/TABLES=Alphablockercontinuationtil1yr BY ASA Brain HTN DM0NO1Yes spinal degenerative disease AURHx ABmonotherapy

@5ARI0NO1Yes B3AAch desmopressin0NO1Yes UDS시행여부No0Yes1

/FORMAT=AVALUE TABLES

/STATISTICS=CHISQ

/CELLS=COUNT ROW COLUMN TOTAL

/COUNT ROUND CELL.

Crosstabs

| **Notes** | | |
| --- | --- | --- |
| Output Created | | 07-JUL-2022 17:10:48 |
| Comments | |  |
| Input | Data | C:\Users\JohnS\Desktop\alpha blocker UDS final raw.sav |
|  | Active Dataset | 데이터세트1 |
|  | Filter | <none> |
|  | Weight | <none> |
|  | Split File | <none> |
|  | N of Rows in Working Data File | 406 |
| Missing Value Handling | Definition of Missing | User-defined missing values are treated as missing. |
|  | Cases Used | Statistics for each table are based on all the cases with valid data in the specified range(s) for all variables in each table. |
| Syntax | | CROSSTABS  /TABLES=Alphablockercontinuationtil1yr BY ASA Brain HTN DM0NO1Yes spinal degenerative disease AURHx ABmonotherapy  @5ARI0NO1Yes B3AAch desmopressin0NO1Yes UDS시행여부No0Yes1  /FORMAT=AVALUE TABLES  /STATISTICS=CHISQ  /CELLS=COUNT ROW COLUMN TOTAL  /COUNT ROUND CELL. |
| Resources | Processor Time | 00:00:00.00 |
|  | Elapsed Time | 00:00:00.01 |
|  | Dimensions Requested | 2 |
|  | Cells Available | 524245 |

| **Warnings** |
| --- |
| The crosstabulation of Alpha blocker continuation til 1yr * AUR Hx is empty. |

| **Case Processing Summary** | | | | | | |
| --- | --- | --- | --- | --- | --- | --- |
|  | Cases | | | | | |
|  | Valid | | Missing | | Total | |
|  | N | Percent | N | Percent | N | Percent |
| Alpha blocker continuation til 1yr * ASA' | 406 | 100.0% | 0 | 0.0% | 406 | 100.0% |
| Alpha blocker continuation til 1yr * Brain | 406 | 100.0% | 0 | 0.0% | 406 | 100.0% |
| Alpha blocker continuation til 1yr * HTN | 406 | 100.0% | 0 | 0.0% | 406 | 100.0% |
| Alpha blocker continuation til 1yr * DM  0: NO  1: Yes | 406 | 100.0% | 0 | 0.0% | 406 | 100.0% |
| Alpha blocker continuation til 1yr * spinal degenerative disease | 406 | 100.0% | 0 | 0.0% | 406 | 100.0% |
| Alpha blocker continuation til 1yr * AB monotherapy | 406 | 100.0% | 0 | 0.0% | 406 | 100.0% |
| Alpha blocker continuation til 1yr * 5ARI  0: NO  1: Yes | 406 | 100.0% | 0 | 0.0% | 406 | 100.0% |
| Alpha blocker continuation til 1yr * B3A +  Ach | 406 | 100.0% | 0 | 0.0% | 406 | 100.0% |
| Alpha blocker continuation til 1yr * desmopressin  0: NO  1: Yes | 406 | 100.0% | 0 | 0.0% | 406 | 100.0% |
| Alpha blocker continuation til 1yr * CONDUCTION OF UDS  No : 0  Yes : 1 | 406 | 100.0% | 0 | 0.0% | 406 | 100.0% |

Alpha blocker continuation til 1yr * ASA'

| **Crosstab** | | | | | | |
| --- | --- | --- | --- | --- | --- | --- |
|  | | | ASA' | | | Total |
|  |  |  | 1 | 2 | 3, 4 |  |
| Alpha blocker continuation til 1yr | 0 | Count | 61 | 175 | 37 | 273 |
|  |  | % within Alpha blocker continuation til 1yr | 22.3% | 64.1% | 13.6% | 100.0% |
|  |  | % within ASA' | 73.5% | 64.3% | 72.5% | 67.2% |
|  |  | % of Total | 15.0% | 43.1% | 9.1% | 67.2% |
|  | 1 | Count | 22 | 97 | 14 | 133 |
|  |  | % within Alpha blocker continuation til 1yr | 16.5% | 72.9% | 10.5% | 100.0% |
|  |  | % within ASA' | 26.5% | 35.7% | 27.5% | 32.8% |
|  |  | % of Total | 5.4% | 23.9% | 3.4% | 32.8% |
| Total | | Count | 83 | 272 | 51 | 406 |
|  |  | % within Alpha blocker continuation til 1yr | 20.4% | 67.0% | 12.6% | 100.0% |
|  |  | % within ASA' | 100.0% | 100.0% | 100.0% | 100.0% |
|  |  | % of Total | 20.4% | 67.0% | 12.6% | 100.0% |

| **Chi-Square Tests** | | | |
| --- | --- | --- | --- |
|  | Value | df | Asymptotic Significance (2-sided) |
| Pearson Chi-Square | 3.166^a^ | 2 | .205 |
| Likelihood Ratio | 3.227 | 2 | .199 |
| Linear-by-Linear Association | .212 | 1 | .645 |
| N of Valid Cases | 406 |  |  |
| a. 0 cells (0.0%) have expected count less than 5. The minimum expected count is 16.71. | | | |

Alpha blocker continuation til 1yr * Brain

| **Crosstab** | | | | | |
| --- | --- | --- | --- | --- | --- |
|  | | | Brain | | Total |
|  |  |  | 0 | 1 |  |
| Alpha blocker continuation til 1yr | 0 | Count | 234 | 39 | 273 |
|  |  | % within Alpha blocker continuation til 1yr | 85.7% | 14.3% | 100.0% |
|  |  | % within Brain | 66.5% | 72.2% | 67.2% |
|  |  | % of Total | 57.6% | 9.6% | 67.2% |
|  | 1 | Count | 118 | 15 | 133 |
|  |  | % within Alpha blocker continuation til 1yr | 88.7% | 11.3% | 100.0% |
|  |  | % within Brain | 33.5% | 27.8% | 32.8% |
|  |  | % of Total | 29.1% | 3.7% | 32.8% |
| Total | | Count | 352 | 54 | 406 |
|  |  | % within Alpha blocker continuation til 1yr | 86.7% | 13.3% | 100.0% |
|  |  | % within Brain | 100.0% | 100.0% | 100.0% |
|  |  | % of Total | 86.7% | 13.3% | 100.0% |

| **Chi-Square Tests** | | | | | |
| --- | --- | --- | --- | --- | --- |
|  | Value | df | Asymptotic Significance (2-sided) | Exact Sig. (2-sided) | Exact Sig. (1-sided) |
| Pearson Chi-Square | .701^a^ | 1 | .402 |  |  |
| Continuity Correction^b^ | .465 | 1 | .495 |  |  |
| Likelihood Ratio | .719 | 1 | .397 |  |  |
| Fisher's Exact Test |  |  |  | .440 | .250 |
| Linear-by-Linear Association | .700 | 1 | .403 |  |  |
| N of Valid Cases | 406 |  |  |  |  |
| a. 0 cells (0.0%) have expected count less than 5. The minimum expected count is 17.69. | | | | | |
| b. Computed only for a 2x2 table | | | | | |

Alpha blocker continuation til 1yr * HTN

| **Crosstab** | | | | | |
| --- | --- | --- | --- | --- | --- |
|  | | | HTN | | Total |
|  |  |  | 0 | 1 |  |
| Alpha blocker continuation til 1yr | 0 | Count | 135 | 138 | 273 |
|  |  | % within Alpha blocker continuation til 1yr | 49.5% | 50.5% | 100.0% |
|  |  | % within HTN | 66.5% | 68.0% | 67.2% |
|  |  | % of Total | 33.3% | 34.0% | 67.2% |
|  | 1 | Count | 68 | 65 | 133 |
|  |  | % within Alpha blocker continuation til 1yr | 51.1% | 48.9% | 100.0% |
|  |  | % within HTN | 33.5% | 32.0% | 32.8% |
|  |  | % of Total | 16.7% | 16.0% | 32.8% |
| Total | | Count | 203 | 203 | 406 |
|  |  | % within Alpha blocker continuation til 1yr | 50.0% | 50.0% | 100.0% |
|  |  | % within HTN | 100.0% | 100.0% | 100.0% |
|  |  | % of Total | 50.0% | 50.0% | 100.0% |

| **Chi-Square Tests** | | | | | |
| --- | --- | --- | --- | --- | --- |
|  | Value | df | Asymptotic Significance (2-sided) | Exact Sig. (2-sided) | Exact Sig. (1-sided) |
| Pearson Chi-Square | .101^a^ | 1 | .751 |  |  |
| Continuity Correction^b^ | .045 | 1 | .833 |  |  |
| Likelihood Ratio | .101 | 1 | .751 |  |  |
| Fisher's Exact Test |  |  |  | .833 | .416 |
| Linear-by-Linear Association | .100 | 1 | .751 |  |  |
| N of Valid Cases | 406 |  |  |  |  |
| a. 0 cells (0.0%) have expected count less than 5. The minimum expected count is 66.50. | | | | | |
| b. Computed only for a 2x2 table | | | | | |

Alpha blocker continuation til 1yr * DM

0: NO

1: Yes

| **Crosstab** | | | | | |
| --- | --- | --- | --- | --- | --- |
|  | | | DM  0: NO  1: Yes | | Total |
|  |  |  | 0 | 1 |  |
| Alpha blocker continuation til 1yr | 0 | Count | 213 | 60 | 273 |
|  |  | % within Alpha blocker continuation til 1yr | 78.0% | 22.0% | 100.0% |
|  |  | % within DM  0: NO  1: Yes | 68.1% | 64.5% | 67.2% |
|  |  | % of Total | 52.5% | 14.8% | 67.2% |
|  | 1 | Count | 100 | 33 | 133 |
|  |  | % within Alpha blocker continuation til 1yr | 75.2% | 24.8% | 100.0% |
|  |  | % within DM  0: NO  1: Yes | 31.9% | 35.5% | 32.8% |
|  |  | % of Total | 24.6% | 8.1% | 32.8% |
| Total | | Count | 313 | 93 | 406 |
|  |  | % within Alpha blocker continuation til 1yr | 77.1% | 22.9% | 100.0% |
|  |  | % within DM  0: NO  1: Yes | 100.0% | 100.0% | 100.0% |
|  |  | % of Total | 77.1% | 22.9% | 100.0% |

| **Chi-Square Tests** | | | | | |
| --- | --- | --- | --- | --- | --- |
|  | Value | df | Asymptotic Significance (2-sided) | Exact Sig. (2-sided) | Exact Sig. (1-sided) |
| Pearson Chi-Square | .407^a^ | 1 | .524 |  |  |
| Continuity Correction^b^ | .262 | 1 | .609 |  |  |
| Likelihood Ratio | .403 | 1 | .526 |  |  |
| Fisher's Exact Test |  |  |  | .531 | .302 |
| Linear-by-Linear Association | .406 | 1 | .524 |  |  |
| N of Valid Cases | 406 |  |  |  |  |
| a. 0 cells (0.0%) have expected count less than 5. The minimum expected count is 30.47. | | | | | |
| b. Computed only for a 2x2 table | | | | | |

Alpha blocker continuation til 1yr * spinal degenerative disease

| **Crosstab** | | | | | |
| --- | --- | --- | --- | --- | --- |
|  | | | spinal degenerative disease | | Total |
|  |  |  | .00 | 1.00 |  |
| Alpha blocker continuation til 1yr | 0 | Count | 245 | 28 | 273 |
|  |  | % within Alpha blocker continuation til 1yr | 89.7% | 10.3% | 100.0% |
|  |  | % within spinal degenerative disease | 68.4% | 58.3% | 67.2% |
|  |  | % of Total | 60.3% | 6.9% | 67.2% |
|  | 1 | Count | 113 | 20 | 133 |
|  |  | % within Alpha blocker continuation til 1yr | 85.0% | 15.0% | 100.0% |
|  |  | % within spinal degenerative disease | 31.6% | 41.7% | 32.8% |
|  |  | % of Total | 27.8% | 4.9% | 32.8% |
| Total | | Count | 358 | 48 | 406 |
|  |  | % within Alpha blocker continuation til 1yr | 88.2% | 11.8% | 100.0% |
|  |  | % within spinal degenerative disease | 100.0% | 100.0% | 100.0% |
|  |  | % of Total | 88.2% | 11.8% | 100.0% |

| **Chi-Square Tests** | | | | | |
| --- | --- | --- | --- | --- | --- |
|  | Value | df | Asymptotic Significance (2-sided) | Exact Sig. (2-sided) | Exact Sig. (1-sided) |
| Pearson Chi-Square | 1.961^a^ | 1 | .161 |  |  |
| Continuity Correction^b^ | 1.529 | 1 | .216 |  |  |
| Likelihood Ratio | 1.896 | 1 | .168 |  |  |
| Fisher's Exact Test |  |  |  | .190 | .109 |
| Linear-by-Linear Association | 1.956 | 1 | .162 |  |  |
| N of Valid Cases | 406 |  |  |  |  |
| a. 0 cells (0.0%) have expected count less than 5. The minimum expected count is 15.72. | | | | | |
| b. Computed only for a 2x2 table | | | | | |

Alpha blocker continuation til 1yr * AUR

| **Crosstab** | | | | | |
| --- | --- | --- | --- | --- | --- |
|  | | | AUR | | Total |
|  |  |  | 0 | 1 |  |
| Alpha blocker continuation til 1yr | 0 | Count | 249 | 24 | 273 |
|  |  | % within Alpha blocker continuation til 1yr | 91.2% | 8.8% | 100.0% |
|  |  | % within AUR | 67.3% | 66.7% | 67.2% |
|  |  | % of Total | 61.3% | 5.9% | 67.2% |
|  | 1 | Count | 121 | 12 | 133 |
|  |  | % within Alpha blocker continuation til 1yr | 91.0% | 9.0% | 100.0% |
|  |  | % within AUR | 32.7% | 33.3% | 32.8% |
|  |  | % of Total | 29.8% | 3.0% | 32.8% |
| Total | | Count | 370 | 36 | 406 |
|  |  | % within Alpha blocker continuation til 1yr | 91.1% | 8.9% | 100.0% |
|  |  | % within AUR | 100.0% | 100.0% | 100.0% |
|  |  | % of Total | 91.1% | 8.9% | 100.0% |

| **Chi-Square Tests** | | | | | |
| --- | --- | --- | --- | --- | --- |
|  | Value | df | Asymptotic Significance (2-sided) | Exact Sig. (2-sided) | Exact Sig. (1-sided) |
| Pearson Chi-Square | .006^a^ | 1 | .939 |  |  |
| Continuity Correction^b^ | .000 | 1 | 1.000 |  |  |
| Likelihood Ratio | .006 | 1 | .939 |  |  |
| Fisher's Exact Test |  |  |  | 1.000 | .536 |
| Linear-by-Linear Association | .006 | 1 | .939 |  |  |
| N of Valid Cases | 406 |  |  |  |  |
| a. 0 cells (0.0%) have expected count less than 5. The minimum expected count is 11.79. | | | | | |
| b. Computed only for a 2x2 table | | | | | |

Alpha blocker continuation til 1yr * AB monotherapy

| **Crosstab** | | | | | |
| --- | --- | --- | --- | --- | --- |
|  | | | AB monotherapy | | Total |
|  |  |  | 0 | 1 |  |
| Alpha blocker continuation til 1yr | 0 | Count | 186 | 87 | 273 |
|  |  | % within Alpha blocker continuation til 1yr | 68.1% | 31.9% | 100.0% |
|  |  | % within AB monotherapy | 69.9% | 62.1% | 67.2% |
|  |  | % of Total | 45.8% | 21.4% | 67.2% |
|  | 1 | Count | 80 | 53 | 133 |
|  |  | % within Alpha blocker continuation til 1yr | 60.2% | 39.8% | 100.0% |
|  |  | % within AB monotherapy | 30.1% | 37.9% | 32.8% |
|  |  | % of Total | 19.7% | 13.1% | 32.8% |
| Total | | Count | 266 | 140 | 406 |
|  |  | % within Alpha blocker continuation til 1yr | 65.5% | 34.5% | 100.0% |
|  |  | % within AB monotherapy | 100.0% | 100.0% | 100.0% |
|  |  | % of Total | 65.5% | 34.5% | 100.0% |

| **Chi-Square Tests** | | | | | |
| --- | --- | --- | --- | --- | --- |
|  | Value | df | Asymptotic Significance (2-sided) | Exact Sig. (2-sided) | Exact Sig. (1-sided) |
| Pearson Chi-Square | 2.522^a^ | 1 | .112 |  |  |
| Continuity Correction^b^ | 2.181 | 1 | .140 |  |  |
| Likelihood Ratio | 2.495 | 1 | .114 |  |  |
| Fisher's Exact Test |  |  |  | .120 | .070 |
| Linear-by-Linear Association | 2.516 | 1 | .113 |  |  |
| N of Valid Cases | 406 |  |  |  |  |
| a. 0 cells (0.0%) have expected count less than 5. The minimum expected count is 45.86. | | | | | |
| b. Computed only for a 2x2 table | | | | | |

Alpha blocker continuation til 1yr * 5ARI

0: NO

1: Yes

| **Crosstab** | | | | | |
| --- | --- | --- | --- | --- | --- |
|  | | | 5ARI  0: NO  1: Yes | | Total |
|  |  |  | 0 | 1 |  |
| Alpha blocker continuation til 1yr | 0 | Count | 121 | 152 | 273 |
|  |  | % within Alpha blocker continuation til 1yr | 44.3% | 55.7% | 100.0% |
|  |  | % within 5ARI  0: NO  1: Yes | 59.9% | 74.5% | 67.2% |
|  |  | % of Total | 29.8% | 37.4% | 67.2% |
|  | 1 | Count | 81 | 52 | 133 |
|  |  | % within Alpha blocker continuation til 1yr | 60.9% | 39.1% | 100.0% |
|  |  | % within 5ARI  0: NO  1: Yes | 40.1% | 25.5% | 32.8% |
|  |  | % of Total | 20.0% | 12.8% | 32.8% |
| Total | | Count | 202 | 204 | 406 |
|  |  | % within Alpha blocker continuation til 1yr | 49.8% | 50.2% | 100.0% |
|  |  | % within 5ARI  0: NO  1: Yes | 100.0% | 100.0% | 100.0% |
|  |  | % of Total | 49.8% | 50.2% | 100.0% |

| **Chi-Square Tests** | | | | | |
| --- | --- | --- | --- | --- | --- |
|  | Value | df | Asymptotic Significance (2-sided) | Exact Sig. (2-sided) | Exact Sig. (1-sided) |
| Pearson Chi-Square | 9.834^a^ | 1 | .002 |  |  |
| Continuity Correction^b^ | 9.182 | 1 | .002 |  |  |
| Likelihood Ratio | 9.892 | 1 | .002 |  |  |
| Fisher's Exact Test |  |  |  | .002 | .001 |
| Linear-by-Linear Association | 9.810 | 1 | .002 |  |  |
| N of Valid Cases | 406 |  |  |  |  |
| a. 0 cells (0.0%) have expected count less than 5. The minimum expected count is 66.17. | | | | | |
| b. Computed only for a 2x2 table | | | | | |

Alpha blocker continuation til 1yr * B3A +

Ach

| **Crosstab** | | | | | |
| --- | --- | --- | --- | --- | --- |
|  | | | B3A +  Ach | | Total |
|  |  |  | 0 | 1 |  |
| Alpha blocker continuation til 1yr | 0 | Count | 207 | 66 | 273 |
|  |  | % within Alpha blocker continuation til 1yr | 75.8% | 24.2% | 100.0% |
|  |  | % within B3A +  Ach | 68.5% | 63.5% | 67.2% |
|  |  | % of Total | 51.0% | 16.3% | 67.2% |
|  | 1 | Count | 95 | 38 | 133 |
|  |  | % within Alpha blocker continuation til 1yr | 71.4% | 28.6% | 100.0% |
|  |  | % within B3A +  Ach | 31.5% | 36.5% | 32.8% |
|  |  | % of Total | 23.4% | 9.4% | 32.8% |
| Total | | Count | 302 | 104 | 406 |
|  |  | % within Alpha blocker continuation til 1yr | 74.4% | 25.6% | 100.0% |
|  |  | % within B3A +  Ach | 100.0% | 100.0% | 100.0% |
|  |  | % of Total | 74.4% | 25.6% | 100.0% |

| **Chi-Square Tests** | | | | | |
| --- | --- | --- | --- | --- | --- |
|  | Value | df | Asymptotic Significance (2-sided) | Exact Sig. (2-sided) | Exact Sig. (1-sided) |
| Pearson Chi-Square | .907^a^ | 1 | .341 |  |  |
| Continuity Correction^b^ | .691 | 1 | .406 |  |  |
| Likelihood Ratio | .896 | 1 | .344 |  |  |
| Fisher's Exact Test |  |  |  | .397 | .202 |
| Linear-by-Linear Association | .905 | 1 | .342 |  |  |
| N of Valid Cases | 406 |  |  |  |  |
| a. 0 cells (0.0%) have expected count less than 5. The minimum expected count is 34.07. | | | | | |
| b. Computed only for a 2x2 table | | | | | |

Alpha blocker continuation til 1yr * desmopressin

0: NO

1: Yes

| **Crosstab** | | | | | |
| --- | --- | --- | --- | --- | --- |
|  | | | desmopressin  0: NO  1: Yes | | Total |
|  |  |  | 0 | 1 |  |
| Alpha blocker continuation til 1yr | 0 | Count | 263 | 10 | 273 |
|  |  | % within Alpha blocker continuation til 1yr | 96.3% | 3.7% | 100.0% |
|  |  | % within desmopressin  0: NO  1: Yes | 67.1% | 71.4% | 67.2% |
|  |  | % of Total | 64.8% | 2.5% | 67.2% |
|  | 1 | Count | 129 | 4 | 133 |
|  |  | % within Alpha blocker continuation til 1yr | 97.0% | 3.0% | 100.0% |
|  |  | % within desmopressin  0: NO  1: Yes | 32.9% | 28.6% | 32.8% |
|  |  | % of Total | 31.8% | 1.0% | 32.8% |
| Total | | Count | 392 | 14 | 406 |
|  |  | % within Alpha blocker continuation til 1yr | 96.6% | 3.4% | 100.0% |
|  |  | % within desmopressin  0: NO  1: Yes | 100.0% | 100.0% | 100.0% |
|  |  | % of Total | 96.6% | 3.4% | 100.0% |

| **Chi-Square Tests** | | | | | |
| --- | --- | --- | --- | --- | --- |
|  | Value | df | Asymptotic Significance (2-sided) | Exact Sig. (2-sided) | Exact Sig. (1-sided) |
| Pearson Chi-Square | .115^a^ | 1 | .734 |  |  |
| Continuity Correction^b^ | .002 | 1 | .960 |  |  |
| Likelihood Ratio | .118 | 1 | .731 |  |  |
| Fisher's Exact Test |  |  |  | 1.000 | .493 |
| Linear-by-Linear Association | .115 | 1 | .734 |  |  |
| N of Valid Cases | 406 |  |  |  |  |
| a. 1 cells (25.0%) have expected count less than 5. The minimum expected count is 4.59. | | | | | |
| b. Computed only for a 2x2 table | | | | | |

Alpha blocker continuation til 1yr * CONDUCTION OF UDS

No : 0

Yes : 1

| **Crosstab** | | | | | |
| --- | --- | --- | --- | --- | --- |
|  | | | CONDUCTION OF UDS  No : 0  Yes : 1 | | Total |
|  |  |  | 0 | 1 |  |
| Alpha blocker continuation til 1yr | 0 | Count | 71 | 202 | 273 |
|  |  | % within Alpha blocker continuation til 1yr | 26.0% | 74.0% | 100.0% |
|  |  | % within CONDUCTION OF UDS  No : 0  Yes : 1 | 46.7% | 79.5% | 67.2% |
|  |  | % of Total | 17.5% | 49.8% | 67.2% |
|  | 1 | Count | 81 | 52 | 133 |
|  |  | % within Alpha blocker continuation til 1yr | 60.9% | 39.1% | 100.0% |
|  |  | % within CONDUCTION OF UDS  No : 0  Yes : 1 | 53.3% | 20.5% | 32.8% |
|  |  | % of Total | 20.0% | 12.8% | 32.8% |
| Total | | Count | 152 | 254 | 406 |
|  |  | % within Alpha blocker continuation til 1yr | 37.4% | 62.6% | 100.0% |
|  |  | % within CONDUCTION OF UDS  No : 0  Yes : 1 | 100.0% | 100.0% | 100.0% |
|  |  | % of Total | 37.4% | 62.6% | 100.0% |

| **Chi-Square Tests** | | | | | |
| --- | --- | --- | --- | --- | --- |
|  | Value | df | Asymptotic Significance (2-sided) | Exact Sig. (2-sided) | Exact Sig. (1-sided) |
| Pearson Chi-Square | 46.493^a^ | 1 | .000 |  |  |
| Continuity Correction^b^ | 45.015 | 1 | .000 |  |  |
| Likelihood Ratio | 46.000 | 1 | .000 |  |  |
| Fisher's Exact Test |  |  |  | .000 | .000 |
| Linear-by-Linear Association | 46.378 | 1 | .000 |  |  |
| N of Valid Cases | 406 |  |  |  |  |
| a. 0 cells (0.0%) have expected count less than 5. The minimum expected count is 49.79. | | | | | |
| b. Computed only for a 2x2 table | | | | | |
