# Supplementary material for: Predictive factors for alpha blocker use after transurethral prostatectomy: Can preoperative urodynamic outcome predict alpha blocker medication after surgery?
Source: PLoS One. 2022 Sep 21;17(9):e0274399. doi: 10.1371/journal.pone.0274399 (PMC9491595; doi:10.1371/journal.pone.0274399)
Supplement: S2 Table — (DOCX) [file pone.0274399.s002.docx]

Logistic Regression

| **Notes** | | |
| --- | --- | --- |
| Output Created | | 07-JUL-2022 22:18:04 |
| Comments | |  |
| Input | Data | C:\Users\JohnS\Desktop\alpha blocker UDS final raw.sav |
|  | Active Dataset | 데이터세트1 |
|  | Filter | <none> |
|  | Weight | <none> |
|  | Split File | <none> |
|  | N of Rows in Working Data File | 406 |
| Missing Value Handling | Definition of Missing | User-defined missing values are treated as missing |
| Syntax | | LOGISTIC REGRESSION VARIABLES Alphablockercontinuationtil1yr  /METHOD=ENTER WHOAge751 duration of taking alpha blockermo @history of preoperative 5-ARI0NO1Yes PreIPSSQ6 Qmax PVR UDS시행여부No0Yes1  /CONTRAST (UDS시행여부No0Yes1)=Indicator  /CONTRAST (@history of preoperative 5-ARI0NO1Yes)=Indicator  /CONTRAST (WHOAge751)=Indicator(1)  /SAVE=LRESID  /PRINT=GOODFIT CI(95)  /CRITERIA=PIN(0.05) POUT(0.10) ITERATE(20) CUT(0.5). |
| Resources | Processor Time | 00:00:00.03 |
|  | Elapsed Time | 00:00:00.02 |
| Variables Created or Modified | LRE_16 | Logit residual |

| **Case Processing Summary** | | | |
| --- | --- | --- | --- |
| Unweighted Cases^a^ | | N | Percent |
| Selected Cases | Included in Analysis | 406 | 100.0 |
|  | Missing Cases | 0 | .0 |
|  | Total | 406 | 100.0 |
| Unselected Cases | | 0 | .0 |
| Total | | 406 | 100.0 |
| a. If weight is in effect, see classification table for the total number of cases. | | | |

| **Dependent Variable Encoding** | |
| --- | --- |
| Original Value | Internal Value |
| 0 | 0 |
| 1 | 1 |

| **Categorical Variables Codings** | | | |
| --- | --- | --- | --- |
|  | | Frequency | Parameter coding |
|  |  |  | (1) |
| CONDUCTION OF UDS  No : 0  Yes : 1 | 0 | 152 | 1.000 |
|  | 1 | 254 | .000 |
| history of preoperative 5-ARI  0: NO  1: Yes | 0 | 202 | 1.000 |
|  | 1 | 204 | .000 |
| WHO  Age>=75 : 1 | 0 | 227 | .000 |
|  | 1 | 179 | 1.000 |

Block 0: Beginning Block

| **Classification Table^a,b^** | | | | | |
| --- | --- | --- | --- | --- | --- |
|  | Observed | | Predicted | | |
|  |  |  | Alpha blocker continuation til 1yr | | Percentage Correct |
|  |  |  | 0 | 1 |  |
| Step 0 | Alpha blocker continuation til 1yr | 0 | 273 | 0 | 100.0 |
|  |  | 1 | 133 | 0 | .0 |
|  | Overall Percentage | |  |  | 67.2 |
| a. Constant is included in the model. | | | | | |
| b. The cut value is .500 | | | | | |

| **Variables in the Equation** | | | | | | | |
| --- | --- | --- | --- | --- | --- | --- | --- |
|  | | B | S.E. | Wald | df | Sig. | Exp(B) |
| Step 0 | Constant | -.719 | .106 | 46.248 | 1 | .000 | .487 |

| **Variables not in the Equation** | | | | | |
| --- | --- | --- | --- | --- | --- |
|  | | | Score | df | Sig. |
| Step 0 | Variables | WHO  Age>=75 : 1(1) | 13.674 | 1 | .000 |
|  |  | duration of taking alpha blocker  (mo) | 6.498 | 1 | .011 |
|  |  | history of preoperative 5-ARI  0: NO  1: Yes(1) | 9.834 | 1 | .002 |
|  |  | Pre IPSS Q6 | 7.387 | 1 | .007 |
|  |  | Qmax | 11.562 | 1 | .001 |
|  |  | PVR | 6.152 | 1 | .013 |
|  |  | CONDUCTION OF UDS  No : 0  Yes : 1(1) | 46.493 | 1 | .000 |
|  | Overall Statistics | | 94.585 | 7 | .000 |

Block 1: Method = Enter

| **Omnibus Tests of Model Coefficients** | | | | |
| --- | --- | --- | --- | --- |
|  | | Chi-square | df | Sig. |
| Step 1 | Step | 104.215 | 7 | .000 |
|  | Block | 104.215 | 7 | .000 |
|  | Model | 104.215 | 7 | .000 |

| **Model Summary** | | | |
| --- | --- | --- | --- |
| Step | -2 Log likelihood | Cox & Snell R Square | Nagelkerke R Square |
| 1 | 409.340^a^ | .226 | .315 |
| a. Estimation terminated at iteration number 5 because parameter estimates changed by less than .001. | | | |

| **Hosmer and Lemeshow Test** | | | |
| --- | --- | --- | --- |
| Step | Chi-square | df | Sig. |
| 1 | 7.865 | 8 | .447 |

| **Contingency Table for Hosmer and Lemeshow Test** | | | | | | |
| --- | --- | --- | --- | --- | --- | --- |
|  | | Alpha blocker continuation til 1yr = 0 | | Alpha blocker continuation til 1yr = 1 | | Total |
|  |  | Observed | Expected | Observed | Expected |  |
| Step 1 | 1 | 39 | 39.014 | 2 | 1.986 | 41 |
|  | 2 | 40 | 37.196 | 1 | 3.804 | 41 |
|  | 3 | 37 | 35.584 | 4 | 5.416 | 41 |
|  | 4 | 36 | 33.598 | 5 | 7.402 | 41 |
|  | 5 | 28 | 31.248 | 13 | 9.752 | 41 |
|  | 6 | 25 | 28.134 | 16 | 12.866 | 41 |
|  | 7 | 22 | 24.453 | 19 | 16.547 | 41 |
|  | 8 | 21 | 20.755 | 20 | 20.245 | 41 |
|  | 9 | 15 | 15.508 | 26 | 25.492 | 41 |
|  | 10 | 10 | 7.511 | 27 | 29.489 | 37 |

| **Classification Table^a^** | | | | | |
| --- | --- | --- | --- | --- | --- |
|  | Observed | | Predicted | | |
|  |  |  | Alpha blocker continuation til 1yr | | Percentage Correct |
|  |  |  | 0 | 1 |  |
| Step 1 | Alpha blocker continuation til 1yr | 0 | 242 | 31 | 88.6 |
|  |  | 1 | 68 | 65 | 48.9 |
|  | Overall Percentage | |  |  | 75.6 |
| a. The cut value is .500 | | | | | |

| **Variables in the Equation** | | | | | | | | | |
| --- | --- | --- | --- | --- | --- | --- | --- | --- | --- |
|  | | B | S.E. | Wald | df | Sig. | Exp(B) | 95% C.I.for EXP(B) | |
|  |  |  |  |  |  |  |  | Lower | Upper |
| Step 1^a^ | WHO  Age>=75 : 1(1) | .902 | .257 | 12.346 | 1 | .000 | 2.463 | 1.490 | 4.073 |
|  | duration of taking alpha blocker  (mo) | .009 | .004 | 5.442 | 1 | .020 | 1.009 | 1.001 | 1.017 |
|  | history of preoperative 5-ARI  0: NO  1: Yes(1) | .782 | .252 | 9.624 | 1 | .002 | 2.186 | 1.334 | 3.583 |
|  | Pre IPSS Q6 | .202 | .069 | 8.657 | 1 | .003 | 1.224 | 1.070 | 1.401 |
|  | Qmax | -.077 | .032 | 5.606 | 1 | .018 | .926 | .869 | .987 |
|  | PVR | .002 | .001 | 5.253 | 1 | .022 | 1.002 | 1.000 | 1.004 |
|  | CONDUCTION OF UDS  No : 0  Yes : 1(1) | 1.803 | .264 | 46.477 | 1 | .000 | 6.067 | 3.613 | 10.188 |
|  | Constant | -2.666 | .515 | 26.800 | 1 | .000 | .069 |  |  |
| a. Variable(s) entered on step 1: WHO  Age>=75 : 1, duration of taking alpha blocker  (mo), history of preoperative 5-ARI  0: NO  1: Yes, Pre IPSS Q6, Qmax, PVR, CONDUCTION OF UDS  No : 0  Yes : 1. | | | | | | | | | |
