# Supplementary material for: Predictive factors for alpha blocker use after transurethral prostatectomy: Can preoperative urodynamic outcome predict alpha blocker medication after surgery?
Source: PLoS One. 2022 Sep 21;17(9):e0274399. doi: 10.1371/journal.pone.0274399 (PMC9491595; doi:10.1371/journal.pone.0274399)
Supplement: S3 Table — (DOCX) [file pone.0274399.s003.docx]

Crosstabs

| **Notes** | | |
| --- | --- | --- |
| Output Created | | 07-JUL-2022 21:31:43 |
| Comments | |  |
| Input | Data | C:\Users\JohnS\Desktop\alpha blocker UDS final raw.sav |
|  | Active Dataset | 데이터세트1 |
|  | Filter | <none> |
|  | Weight | <none> |
|  | Split File | <none> |
|  | N of Rows in Working Data File | 406 |
| Missing Value Handling | Definition of Missing | User-defined missing values are treated as missing. |
|  | Cases Used | Statistics for each table are based on all the cases with valid data in the specified range(s) for all variables in each table. |
| Syntax | | CROSSTABS  /TABLES=B1DB2D3 BY alphablocker0NO1Yes_A alphablocker0NO1Yes_B alphablocker0NO1Yes_C  alphablocker0NO1Yes_D Alphablockercontinuationtil1yr  /FORMAT=AVALUE TABLES  /STATISTICS=CHISQ  /CELLS=COUNT ROW COLUMN TOTAL  /COUNT ROUND CELL. |
| Resources | Processor Time | 00:00:00.02 |
|  | Elapsed Time | 00:00:00.01 |
|  | Dimensions Requested | 2 |
|  | Cells Available | 524245 |

| **Case Processing Summary** | | | | | | |
| --- | --- | --- | --- | --- | --- | --- |
|  | Cases | | | | | |
|  | Valid | | Missing | | Total | |
|  | N | Percent | N | Percent | N | Percent |
| B1 D+B2 D3 * alpha-blocker  0: NO  1: Yes | 254 | 62.6% | 152 | 37.4% | 406 | 100.0% |
| B1 D+B2 D3 * alpha-blocker  0: NO  1: Yes | 254 | 62.6% | 152 | 37.4% | 406 | 100.0% |
| B1 D+B2 D3 * alpha-blocker  0: NO  1: Yes | 254 | 62.6% | 152 | 37.4% | 406 | 100.0% |
| B1 D+B2 D3 * alpha-blocker  0: NO  1: Yes | 254 | 62.6% | 152 | 37.4% | 406 | 100.0% |
| B1 D+B2 D3 * Alpha blocker continuation til 1yr | 254 | 62.6% | 152 | 37.4% | 406 | 100.0% |

B1 D+B2 D3 * alpha-blocker 1month

0: NO

1: Yes

| **Crosstab** | | | | | |
| --- | --- | --- | --- | --- | --- |
|  | | | alpha-blocker  0: NO  1: Yes | | Total |
|  |  |  | 0 | 1 |  |
| B1 D+B2 D3 | 0 | Count | 15 | 16 | 31 |
|  |  | % within B1 D+B2 D3 | 48.4% | 51.6% | 100.0% |
|  |  | % within alpha-blocker  0: NO  1: Yes | 15.3% | 10.3% | 12.2% |
|  |  | % of Total | 5.9% | 6.3% | 12.2% |
|  | 1 | Count | 43 | 85 | 128 |
|  |  | % within B1 D+B2 D3 | 33.6% | 66.4% | 100.0% |
|  |  | % within alpha-blocker  0: NO  1: Yes | 43.9% | 54.5% | 50.4% |
|  |  | % of Total | 16.9% | 33.5% | 50.4% |
|  | 2 | Count | 20 | 24 | 44 |
|  |  | % within B1 D+B2 D3 | 45.5% | 54.5% | 100.0% |
|  |  | % within alpha-blocker  0: NO  1: Yes | 20.4% | 15.4% | 17.3% |
|  |  | % of Total | 7.9% | 9.4% | 17.3% |
|  | 3 | Count | 20 | 31 | 51 |
|  |  | % within B1 D+B2 D3 | 39.2% | 60.8% | 100.0% |
|  |  | % within alpha-blocker  0: NO  1: Yes | 20.4% | 19.9% | 20.1% |
|  |  | % of Total | 7.9% | 12.2% | 20.1% |
| Total | | Count | 98 | 156 | 254 |
|  |  | % within B1 D+B2 D3 | 38.6% | 61.4% | 100.0% |
|  |  | % within alpha-blocker  0: NO  1: Yes | 100.0% | 100.0% | 100.0% |
|  |  | % of Total | 38.6% | 61.4% | 100.0% |

| **Chi-Square Tests** | | | |
| --- | --- | --- | --- |
|  | Value | df | Asymptotic Significance (2-sided) |
| Pearson Chi-Square | 3.487^a^ | 3 | .322 |
| Likelihood Ratio | 3.466 | 3 | .325 |
| Linear-by-Linear Association | .007 | 1 | .932 |
| N of Valid Cases | 254 |  |  |
| a. 0 cells (0.0%) have expected count less than 5. The minimum expected count is 11.96. | | | |

B1 D+B2 D3 * alpha-blocker 3 months

0: NO

1: Yes

| **Crosstab** | | | | | |
| --- | --- | --- | --- | --- | --- |
|  | | | alpha-blocker  0: NO  1: Yes | | Total |
|  |  |  | 0 | 1 |  |
| B1 D+B2 D3 | 0 | Count | 25 | 6 | 31 |
|  |  | % within B1 D+B2 D3 | 80.6% | 19.4% | 100.0% |
|  |  | % within alpha-blocker  0: NO  1: Yes | 16.2% | 6.0% | 12.2% |
|  |  | % of Total | 9.8% | 2.4% | 12.2% |
|  | 1 | Count | 70 | 58 | 128 |
|  |  | % within B1 D+B2 D3 | 54.7% | 45.3% | 100.0% |
|  |  | % within alpha-blocker  0: NO  1: Yes | 45.5% | 58.0% | 50.4% |
|  |  | % of Total | 27.6% | 22.8% | 50.4% |
|  | 2 | Count | 30 | 14 | 44 |
|  |  | % within B1 D+B2 D3 | 68.2% | 31.8% | 100.0% |
|  |  | % within alpha-blocker  0: NO  1: Yes | 19.5% | 14.0% | 17.3% |
|  |  | % of Total | 11.8% | 5.5% | 17.3% |
|  | 3 | Count | 29 | 22 | 51 |
|  |  | % within B1 D+B2 D3 | 56.9% | 43.1% | 100.0% |
|  |  | % within alpha-blocker  0: NO  1: Yes | 18.8% | 22.0% | 20.1% |
|  |  | % of Total | 11.4% | 8.7% | 20.1% |
| Total | | Count | 154 | 100 | 254 |
|  |  | % within B1 D+B2 D3 | 60.6% | 39.4% | 100.0% |
|  |  | % within alpha-blocker  0: NO  1: Yes | 100.0% | 100.0% | 100.0% |
|  |  | % of Total | 60.6% | 39.4% | 100.0% |

| **Chi-Square Tests** | | | |
| --- | --- | --- | --- |
|  | Value | df | Asymptotic Significance (2-sided) |
| Pearson Chi-Square | 8.451^a^ | 3 | .038 |
| Likelihood Ratio | 8.989 | 3 | .029 |
| Linear-by-Linear Association | .831 | 1 | .362 |
| N of Valid Cases | 254 |  |  |
| a. 0 cells (0.0%) have expected count less than 5. The minimum expected count is 12.20. | | | |

B1 D+B2 D3 * alpha-blocker 6 months

0: NO

1: Yes

| **Crosstab** | | | | | |
| --- | --- | --- | --- | --- | --- |
|  | | | alpha-blocker  0: NO  1: Yes | | Total |
|  |  |  | 0 | 1 |  |
| B1 D+B2 D3 | 0 | Count | 27 | 4 | 31 |
|  |  | % within B1 D+B2 D3 | 87.1% | 12.9% | 100.0% |
|  |  | % within alpha-blocker  0: NO  1: Yes | 14.9% | 5.5% | 12.2% |
|  |  | % of Total | 10.6% | 1.6% | 12.2% |
|  | 1 | Count | 82 | 46 | 128 |
|  |  | % within B1 D+B2 D3 | 64.1% | 35.9% | 100.0% |
|  |  | % within alpha-blocker  0: NO  1: Yes | 45.3% | 63.0% | 50.4% |
|  |  | % of Total | 32.3% | 18.1% | 50.4% |
|  | 2 | Count | 36 | 8 | 44 |
|  |  | % within B1 D+B2 D3 | 81.8% | 18.2% | 100.0% |
|  |  | % within alpha-blocker  0: NO  1: Yes | 19.9% | 11.0% | 17.3% |
|  |  | % of Total | 14.2% | 3.1% | 17.3% |
|  | 3 | Count | 36 | 15 | 51 |
|  |  | % within B1 D+B2 D3 | 70.6% | 29.4% | 100.0% |
|  |  | % within alpha-blocker  0: NO  1: Yes | 19.9% | 20.5% | 20.1% |
|  |  | % of Total | 14.2% | 5.9% | 20.1% |
| Total | | Count | 181 | 73 | 254 |
|  |  | % within B1 D+B2 D3 | 71.3% | 28.7% | 100.0% |
|  |  | % within alpha-blocker  0: NO  1: Yes | 100.0% | 100.0% | 100.0% |
|  |  | % of Total | 71.3% | 28.7% | 100.0% |

| **Chi-Square Tests** | | | |
| --- | --- | --- | --- |
|  | Value | df | Asymptotic Significance (2-sided) |
| Pearson Chi-Square | 9.440^a^ | 3 | .024 |
| Likelihood Ratio | 10.163 | 3 | .017 |
| Linear-by-Linear Association | .019 | 1 | .890 |
| N of Valid Cases | 254 |  |  |
| a. 0 cells (0.0%) have expected count less than 5. The minimum expected count is 8.91. | | | |

B1 D+B2 D3 * alpha-blocker 12months

0: NO

1: Yes

| **Crosstab** | | | | | |
| --- | --- | --- | --- | --- | --- |
|  | | | alpha-blocker  0: NO  1: Yes | | Total |
|  |  |  | 0 | 1 |  |
| B1 D+B2 D3 | 0 | Count | 26 | 5 | 31 |
|  |  | % within B1 D+B2 D3 | 83.9% | 16.1% | 100.0% |
|  |  | % within alpha-blocker  0: NO  1: Yes | 15.0% | 6.2% | 12.2% |
|  |  | % of Total | 10.2% | 2.0% | 12.2% |
|  | 1 | Count | 74 | 54 | 128 |
|  |  | % within B1 D+B2 D3 | 57.8% | 42.2% | 100.0% |
|  |  | % within alpha-blocker  0: NO  1: Yes | 42.8% | 66.7% | 50.4% |
|  |  | % of Total | 29.1% | 21.3% | 50.4% |
|  | 2 | Count | 38 | 6 | 44 |
|  |  | % within B1 D+B2 D3 | 86.4% | 13.6% | 100.0% |
|  |  | % within alpha-blocker  0: NO  1: Yes | 22.0% | 7.4% | 17.3% |
|  |  | % of Total | 15.0% | 2.4% | 17.3% |
|  | 3 | Count | 35 | 16 | 51 |
|  |  | % within B1 D+B2 D3 | 68.6% | 31.4% | 100.0% |
|  |  | % within alpha-blocker  0: NO  1: Yes | 20.2% | 19.8% | 20.1% |
|  |  | % of Total | 13.8% | 6.3% | 20.1% |
| Total | | Count | 173 | 81 | 254 |
|  |  | % within B1 D+B2 D3 | 68.1% | 31.9% | 100.0% |
|  |  | % within alpha-blocker  0: NO  1: Yes | 100.0% | 100.0% | 100.0% |
|  |  | % of Total | 68.1% | 31.9% | 100.0% |

| **Chi-Square Tests** | | | |
| --- | --- | --- | --- |
|  | Value | df | Asymptotic Significance (2-sided) |
| Pearson Chi-Square | 16.550^a^ | 3 | .001 |
| Likelihood Ratio | 17.826 | 3 | .000 |
| Linear-by-Linear Association | .273 | 1 | .602 |
| N of Valid Cases | 254 |  |  |
| a. 0 cells (0.0%) have expected count less than 5. The minimum expected count is 9.89. | | | |

B1 D+B2 D3 * Alpha blocker continuation until 1yr

| **Crosstab** | | | | | |
| --- | --- | --- | --- | --- | --- |
|  | | | Alpha blocker continuation til 1yr | | Total |
|  |  |  | 0 | 1 |  |
| B1 D+B2 D3 | 0 | Count | 27 | 4 | 31 |
|  |  | % within B1 D+B2 D3 | 87.1% | 12.9% | 100.0% |
|  |  | % within Alpha blocker continuation til 1yr | 13.4% | 7.7% | 12.2% |
|  |  | % of Total | 10.6% | 1.6% | 12.2% |
|  | 1 | Count | 93 | 35 | 128 |
|  |  | % within B1 D+B2 D3 | 72.7% | 27.3% | 100.0% |
|  |  | % within Alpha blocker continuation til 1yr | 46.0% | 67.3% | 50.4% |
|  |  | % of Total | 36.6% | 13.8% | 50.4% |
|  | 2 | Count | 41 | 3 | 44 |
|  |  | % within B1 D+B2 D3 | 93.2% | 6.8% | 100.0% |
|  |  | % within Alpha blocker continuation til 1yr | 20.3% | 5.8% | 17.3% |
|  |  | % of Total | 16.1% | 1.2% | 17.3% |
|  | 3 | Count | 41 | 10 | 51 |
|  |  | % within B1 D+B2 D3 | 80.4% | 19.6% | 100.0% |
|  |  | % within Alpha blocker continuation til 1yr | 20.3% | 19.2% | 20.1% |
|  |  | % of Total | 16.1% | 3.9% | 20.1% |
| Total | | Count | 202 | 52 | 254 |
|  |  | % within B1 D+B2 D3 | 79.5% | 20.5% | 100.0% |
|  |  | % within Alpha blocker continuation til 1yr | 100.0% | 100.0% | 100.0% |
|  |  | % of Total | 79.5% | 20.5% | 100.0% |

| **Chi-Square Tests** | | | |
| --- | --- | --- | --- |
|  | Value | df | Asymptotic Significance (2-sided) |
| Pearson Chi-Square | 9.865^a^ | 3 | .020 |
| Likelihood Ratio | 11.087 | 3 | .011 |
| Linear-by-Linear Association | .556 | 1 | .456 |
| N of Valid Cases | 254 |  |  |
| a. 0 cells (0.0%) have expected count less than 5. The minimum expected count is 6.35. | | | |

Crosstabs

| **Notes** | | |
| --- | --- | --- |
| Output Created | | 07-JUL-2022 21:32:26 |
| Comments | |  |
| Input | Data | C:\Users\JohnS\Desktop\alpha blocker UDS final raw.sav |
|  | Active Dataset | 데이터세트1 |
|  | Filter | <none> |
|  | Weight | <none> |
|  | Split File | <none> |
|  | N of Rows in Working Data File | 406 |
| Missing Value Handling | Definition of Missing | User-defined missing values are treated as missing. |
|  | Cases Used | Statistics for each table are based on all the cases with valid data in the specified range(s) for all variables in each table. |
| Syntax | | CROSSTABS  /TABLES=UDS시행여부No0Yes1 BY alphablocker0NO1Yes_A alphablocker0NO1Yes_B alphablocker0NO1Yes_C  alphablocker0NO1Yes_D Alphablockercontinuationtil1yr  /FORMAT=AVALUE TABLES  /STATISTICS=CHISQ  /CELLS=COUNT ROW COLUMN TOTAL  /COUNT ROUND CELL. |
| Resources | Processor Time | 00:00:00.02 |
|  | Elapsed Time | 00:00:00.01 |
|  | Dimensions Requested | 2 |
|  | Cells Available | 524245 |

| **Case Processing Summary** | | | | | | |
| --- | --- | --- | --- | --- | --- | --- |
|  | Cases | | | | | |
|  | Valid | | Missing | | Total | |
|  | N | Percent | N | Percent | N | Percent |
| CONDUCTION OF UDS  No : 0  Yes : 1 * alpha-blocker  0: NO  1: Yes | 406 | 100.0% | 0 | 0.0% | 406 | 100.0% |
| CONDUCTION OF UDS  No : 0  Yes : 1 * alpha-blocker  0: NO  1: Yes | 406 | 100.0% | 0 | 0.0% | 406 | 100.0% |
| CONDUCTION OF UDS  No : 0  Yes : 1 * alpha-blocker  0: NO  1: Yes | 406 | 100.0% | 0 | 0.0% | 406 | 100.0% |
| CONDUCTION OF UDS  No : 0  Yes : 1 * alpha-blocker  0: NO  1: Yes | 406 | 100.0% | 0 | 0.0% | 406 | 100.0% |
| CONDUCTION OF UDS  No : 0  Yes : 1 * Alpha blocker continuation til 1yr | 406 | 100.0% | 0 | 0.0% | 406 | 100.0% |

CONDUCTION OF UDS

No : 0

Yes : 1 * alpha-blocker 1 month

0: NO

1: Yes

| **Crosstab** | | | | | |
| --- | --- | --- | --- | --- | --- |
|  | | | alpha-blocker  0: NO  1: Yes | | Total |
|  |  |  | 0 | 1 |  |
| CONDUCTION OF UDS  No : 0  Yes : 1 | 0 | Count | 27 | 125 | 152 |
|  |  | % within CONDUCTION OF UDS  No : 0  Yes : 1 | 17.8% | 82.2% | 100.0% |
|  |  | % within alpha-blocker  0: NO  1: Yes | 21.6% | 44.5% | 37.4% |
|  |  | % of Total | 6.7% | 30.8% | 37.4% |
|  | 1 | Count | 98 | 156 | 254 |
|  |  | % within CONDUCTION OF UDS  No : 0  Yes : 1 | 38.6% | 61.4% | 100.0% |
|  |  | % within alpha-blocker  0: NO  1: Yes | 78.4% | 55.5% | 62.6% |
|  |  | % of Total | 24.1% | 38.4% | 62.6% |
| Total | | Count | 125 | 281 | 406 |
|  |  | % within CONDUCTION OF UDS  No : 0  Yes : 1 | 30.8% | 69.2% | 100.0% |
|  |  | % within alpha-blocker  0: NO  1: Yes | 100.0% | 100.0% | 100.0% |
|  |  | % of Total | 30.8% | 69.2% | 100.0% |

| **Chi-Square Tests** | | | | | |
| --- | --- | --- | --- | --- | --- |
|  | Value | df | Asymptotic Significance (2-sided) | Exact Sig. (2-sided) | Exact Sig. (1-sided) |
| Pearson Chi-Square | 19.343^a^ | 1 | .000 |  |  |
| Continuity Correction^b^ | 18.379 | 1 | .000 |  |  |
| Likelihood Ratio | 20.362 | 1 | .000 |  |  |
| Fisher's Exact Test |  |  |  | .000 | .000 |
| Linear-by-Linear Association | 19.296 | 1 | .000 |  |  |
| N of Valid Cases | 406 |  |  |  |  |
| a. 0 cells (0.0%) have expected count less than 5. The minimum expected count is 46.80. | | | | | |
| b. Computed only for a 2x2 table | | | | | |

CONDUCTION OF UDS

No : 0

Yes : 1 * alpha-blocker 3 months

0: NO

1: Yes

| **Crosstab** | | | | | |
| --- | --- | --- | --- | --- | --- |
|  | | | alpha-blocker  0: NO  1: Yes | | Total |
|  |  |  | 0 | 1 |  |
| CONDUCTION OF UDS  No : 0  Yes : 1 | 0 | Count | 28 | 124 | 152 |
|  |  | % within CONDUCTION OF UDS  No : 0  Yes : 1 | 18.4% | 81.6% | 100.0% |
|  |  | % within alpha-blocker  0: NO  1: Yes | 15.4% | 55.4% | 37.4% |
|  |  | % of Total | 6.9% | 30.5% | 37.4% |
|  | 1 | Count | 154 | 100 | 254 |
|  |  | % within CONDUCTION OF UDS  No : 0  Yes : 1 | 60.6% | 39.4% | 100.0% |
|  |  | % within alpha-blocker  0: NO  1: Yes | 84.6% | 44.6% | 62.6% |
|  |  | % of Total | 37.9% | 24.6% | 62.6% |
| Total | | Count | 182 | 224 | 406 |
|  |  | % within CONDUCTION OF UDS  No : 0  Yes : 1 | 44.8% | 55.2% | 100.0% |
|  |  | % within alpha-blocker  0: NO  1: Yes | 100.0% | 100.0% | 100.0% |
|  |  | % of Total | 44.8% | 55.2% | 100.0% |

| **Chi-Square Tests** | | | | | |
| --- | --- | --- | --- | --- | --- |
|  | Value | df | Asymptotic Significance (2-sided) | Exact Sig. (2-sided) | Exact Sig. (1-sided) |
| Pearson Chi-Square | 68.500^a^ | 1 | .000 |  |  |
| Continuity Correction^b^ | 66.804 | 1 | .000 |  |  |
| Likelihood Ratio | 72.706 | 1 | .000 |  |  |
| Fisher's Exact Test |  |  |  | .000 | .000 |
| Linear-by-Linear Association | 68.331 | 1 | .000 |  |  |
| N of Valid Cases | 406 |  |  |  |  |
| a. 0 cells (0.0%) have expected count less than 5. The minimum expected count is 68.14. | | | | | |
| b. Computed only for a 2x2 table | | | | | |

CONDUCTION OF UDS

No : 0

Yes : 1 * alpha-blocker 6 months

0: NO

1: Yes

| **Crosstab** | | | | | |
| --- | --- | --- | --- | --- | --- |
|  | | | alpha-blocker  0: NO  1: Yes | | Total |
|  |  |  | 0 | 1 |  |
| CONDUCTION OF UDS  No : 0  Yes : 1 | 0 | Count | 50 | 102 | 152 |
|  |  | % within CONDUCTION OF UDS  No : 0  Yes : 1 | 32.9% | 67.1% | 100.0% |
|  |  | % within alpha-blocker  0: NO  1: Yes | 21.6% | 58.3% | 37.4% |
|  |  | % of Total | 12.3% | 25.1% | 37.4% |
|  | 1 | Count | 181 | 73 | 254 |
|  |  | % within CONDUCTION OF UDS  No : 0  Yes : 1 | 71.3% | 28.7% | 100.0% |
|  |  | % within alpha-blocker  0: NO  1: Yes | 78.4% | 41.7% | 62.6% |
|  |  | % of Total | 44.6% | 18.0% | 62.6% |
| Total | | Count | 231 | 175 | 406 |
|  |  | % within CONDUCTION OF UDS  No : 0  Yes : 1 | 56.9% | 43.1% | 100.0% |
|  |  | % within alpha-blocker  0: NO  1: Yes | 100.0% | 100.0% | 100.0% |
|  |  | % of Total | 56.9% | 43.1% | 100.0% |

| **Chi-Square Tests** | | | | | |
| --- | --- | --- | --- | --- | --- |
|  | Value | df | Asymptotic Significance (2-sided) | Exact Sig. (2-sided) | Exact Sig. (1-sided) |
| Pearson Chi-Square | 57.072^a^ | 1 | .000 |  |  |
| Continuity Correction^b^ | 55.519 | 1 | .000 |  |  |
| Likelihood Ratio | 57.821 | 1 | .000 |  |  |
| Fisher's Exact Test |  |  |  | .000 | .000 |
| Linear-by-Linear Association | 56.932 | 1 | .000 |  |  |
| N of Valid Cases | 406 |  |  |  |  |
| a. 0 cells (0.0%) have expected count less than 5. The minimum expected count is 65.52. | | | | | |
| b. Computed only for a 2x2 table | | | | | |

CONDUCTION OF UDS

No : 0

Yes : 1 * alpha-blocker 12 months

0: NO

1: Yes

| **Crosstab** | | | | | |
| --- | --- | --- | --- | --- | --- |
|  | | | alpha-blocker  0: NO  1: Yes | | Total |
|  |  |  | 0 | 1 |  |
| CONDUCTION OF UDS  No : 0  Yes : 1 | 0 | Count | 54 | 98 | 152 |
|  |  | % within CONDUCTION OF UDS  No : 0  Yes : 1 | 35.5% | 64.5% | 100.0% |
|  |  | % within alpha-blocker  0: NO  1: Yes | 23.8% | 54.7% | 37.4% |
|  |  | % of Total | 13.3% | 24.1% | 37.4% |
|  | 1 | Count | 173 | 81 | 254 |
|  |  | % within CONDUCTION OF UDS  No : 0  Yes : 1 | 68.1% | 31.9% | 100.0% |
|  |  | % within alpha-blocker  0: NO  1: Yes | 76.2% | 45.3% | 62.6% |
|  |  | % of Total | 42.6% | 20.0% | 62.6% |
| Total | | Count | 227 | 179 | 406 |
|  |  | % within CONDUCTION OF UDS  No : 0  Yes : 1 | 55.9% | 44.1% | 100.0% |
|  |  | % within alpha-blocker  0: NO  1: Yes | 100.0% | 100.0% | 100.0% |
|  |  | % of Total | 55.9% | 44.1% | 100.0% |

| **Chi-Square Tests** | | | | | |
| --- | --- | --- | --- | --- | --- |
|  | Value | df | Asymptotic Significance (2-sided) | Exact Sig. (2-sided) | Exact Sig. (1-sided) |
| Pearson Chi-Square | 40.957^a^ | 1 | .000 |  |  |
| Continuity Correction^b^ | 39.646 | 1 | .000 |  |  |
| Likelihood Ratio | 41.325 | 1 | .000 |  |  |
| Fisher's Exact Test |  |  |  | .000 | .000 |
| Linear-by-Linear Association | 40.856 | 1 | .000 |  |  |
| N of Valid Cases | 406 |  |  |  |  |
| a. 0 cells (0.0%) have expected count less than 5. The minimum expected count is 67.01. | | | | | |
| b. Computed only for a 2x2 table | | | | | |

CONDUCTION OF UDS

No : 0

Yes : 1 * Alpha blocker continuation until 1yr

| **Crosstab** | | | | | |
| --- | --- | --- | --- | --- | --- |
|  | | | Alpha blocker continuation til 1yr | | Total |
|  |  |  | 0 | 1 |  |
| CONDUCTION OF UDS  No : 0  Yes : 1 | 0 | Count | 71 | 81 | 152 |
|  |  | % within CONDUCTION OF UDS  No : 0  Yes : 1 | 46.7% | 53.3% | 100.0% |
|  |  | % within Alpha blocker continuation til 1yr | 26.0% | 60.9% | 37.4% |
|  |  | % of Total | 17.5% | 20.0% | 37.4% |
|  | 1 | Count | 202 | 52 | 254 |
|  |  | % within CONDUCTION OF UDS  No : 0  Yes : 1 | 79.5% | 20.5% | 100.0% |
|  |  | % within Alpha blocker continuation til 1yr | 74.0% | 39.1% | 62.6% |
|  |  | % of Total | 49.8% | 12.8% | 62.6% |
| Total | | Count | 273 | 133 | 406 |
|  |  | % within CONDUCTION OF UDS  No : 0  Yes : 1 | 67.2% | 32.8% | 100.0% |
|  |  | % within Alpha blocker continuation til 1yr | 100.0% | 100.0% | 100.0% |
|  |  | % of Total | 67.2% | 32.8% | 100.0% |

| **Chi-Square Tests** | | | | | |
| --- | --- | --- | --- | --- | --- |
|  | Value | df | Asymptotic Significance (2-sided) | Exact Sig. (2-sided) | Exact Sig. (1-sided) |
| Pearson Chi-Square | 46.493^a^ | 1 | .000 |  |  |
| Continuity Correction^b^ | 45.015 | 1 | .000 |  |  |
| Likelihood Ratio | 46.000 | 1 | .000 |  |  |
| Fisher's Exact Test |  |  |  | .000 | .000 |
| Linear-by-Linear Association | 46.378 | 1 | .000 |  |  |
| N of Valid Cases | 406 |  |  |  |  |
| a. 0 cells (0.0%) have expected count less than 5. The minimum expected count is 49.79. | | | | | |
| b. Computed only for a 2x2 table | | | | | |
