# Supplementary material for: Predictive factors for alpha blocker use after transurethral prostatectomy: Can preoperative urodynamic outcome predict alpha blocker medication after surgery?
Source: PLoS One. 2022 Sep 21;17(9):e0274399. doi: 10.1371/journal.pone.0274399 (PMC9491595; doi:10.1371/journal.pone.0274399)
Supplement: S4 Table — (DOCX) [file pone.0274399.s004.docx]

T-Test

| **Notes** | | |
| --- | --- | --- |
| Output Created | | 07-JUL-2022 21:29:01 |
| Comments | |  |
| Input | Data | C:\Users\JohnS\Desktop\alpha blocker UDS final raw.sav |
|  | Active Dataset | 데이터세트1 |
|  | Filter | <none> |
|  | Weight | <none> |
|  | Split File | <none> |
|  | N of Rows in Working Data File | 406 |
| Missing Value Handling | Definition of Missing | User defined missing values are treated as missing. |
|  | Cases Used | Statistics for each analysis are based on the cases with no missing or out-of-range data for any variable in the analysis. |
| Syntax | | T-TEST GROUPS=Alphablockercontinuationtil1yr(0 1)  /MISSING=ANALYSIS  /VARIABLES=VS SS T T_A PostIPSSQ8_D deltaIPSSQ8 Qmax_F deltaQ PVR_E deltaP  /ES DISPLAY(TRUE)  /CRITERIA=CI(.95). |
| Resources | Processor Time | 00:00:00.02 |
|  | Elapsed Time | 00:00:00.02 |

| **Group Statistics** | | | | | |
| --- | --- | --- | --- | --- | --- |
|  | Alpha blocker continuation til 1yr | N | Mean | Std. Deviation | Std. Error Mean |
| VS | 0 | 273 | 4.19 | 4.378 | .265 |
|  | 1 | 133 | 5.55 | 5.370 | .466 |
| SS | 0 | 273 | 4.24 | 3.157 | .191 |
|  | 1 | 133 | 4.80 | 3.339 | .290 |
| Total | 0 | 273 | 8.43 | 6.740 | .408 |
|  | 1 | 133 | 10.35 | 7.963 | .690 |
| Delta Total | 0 | 273 | 10.24 | 8.277 | .501 |
|  | 1 | 133 | 10.62 | 8.207 | .712 |
| Post IPSS Q8 | 0 | 273 | 2.50 | 1.304 | .079 |
|  | 1 | 133 | 2.48 | 1.335 | .116 |
| delta IPSS Q8 | 0 | 273 | 1.75 | 1.462 | .088 |
|  | 1 | 133 | 1.89 | 1.682 | .146 |
| Qmax' | 0 | 273 | 18.48 | 8.752 | .530 |
|  | 1 | 133 | 15.98 | 7.508 | .651 |
| Delta Qmax | 0 | 273 | -8.960073260073267 | 8.015351722158382 | .485111154627455 |
|  | 1 | 133 | -8.027067669172931 | 7.937522396241836 | .688270480310228 |
| PVR' | 0 | 273 | 29.34 | 25.109 | 1.520 |
|  | 1 | 133 | 40.68 | 24.563 | 2.130 |
| Delta PVR | 0 | 273 | 59.141 | 102.3131 | 6.1923 |
|  | 1 | 133 | 79.744 | 155.7833 | 13.5081 |

| **Independent Samples Test** | | | | | | | | | | |
| --- | --- | --- | --- | --- | --- | --- | --- | --- | --- | --- |
|  | | Levene's Test for Equality of Variances | | t-test for Equality of Means | | | | | | |
|  |  | F | Sig. | t | df | Sig. (2-tailed) | Mean Difference | Std. Error Difference | 95% Confidence Interval of the Difference | |
|  |  |  |  |  |  |  |  |  | Lower | Upper |
| VS | Equal variances assumed | 10.278 | .001 | -2.711 | 404 | .007 | -1.355 | .500 | -2.337 | -.373 |
|  | Equal variances not assumed |  |  | -2.529 | 220.104 | .012 | -1.355 | .536 | -2.411 | -.299 |
| SS | Equal variances assumed | .510 | .476 | -1.643 | 404 | .101 | -.559 | .340 | -1.228 | .110 |
|  | Equal variances not assumed |  |  | -1.611 | 249.120 | .108 | -.559 | .347 | -1.242 | .124 |
| Total | Equal variances assumed | 6.922 | .009 | -2.527 | 404 | .012 | -1.914 | .757 | -3.403 | -.425 |
|  | Equal variances not assumed |  |  | -2.386 | 226.803 | .018 | -1.914 | .802 | -3.494 | -.333 |
| Delta Total | Equal variances assumed | .890 | .346 | -.434 | 404 | .665 | -.378 | .873 | -2.094 | 1.337 |
|  | Equal variances not assumed |  |  | -.435 | 263.796 | .664 | -.378 | .870 | -2.092 | 1.335 |
| Post IPSS Q8 | Equal variances assumed | .000 | .997 | .148 | 404 | .882 | .021 | .139 | -.253 | .294 |
|  | Equal variances not assumed |  |  | .147 | 256.312 | .883 | .021 | .140 | -.255 | .296 |
| delta IPSS Q8 | Equal variances assumed | 5.057 | .025 | -.839 | 404 | .402 | -.136 | .163 | -.456 | .183 |
|  | Equal variances not assumed |  |  | -.799 | 231.795 | .425 | -.136 | .171 | -.472 | .200 |
| Qmax | Equal variances assumed | 6.709 | .010 | 2.833 | 404 | .005 | 2.506 | .885 | .767 | 4.245 |
|  | Equal variances not assumed |  |  | 2.986 | 300.666 | .003 | 2.506 | .839 | .854 | 4.158 |
| Delta Qmax | Equal variances assumed | .915 | .339 | -1.104 | 404 | .270 | -.933005590900336 | .844895434272473 | -2.593946047293816 | .727934865493144 |
|  | Equal variances not assumed |  |  | -1.108 | 264.097 | .269 | -.933005590900336 | .842050524856113 | -2.590992281649979 | .724981099849306 |
| PVR' | Equal variances assumed | .361 | .548 | -4.300 | 404 | .000 | -11.336 | 2.636 | -16.519 | -6.153 |
|  | Equal variances not assumed |  |  | -4.333 | 267.023 | .000 | -11.336 | 2.616 | -16.487 | -6.185 |
| Delta PVR | Equal variances assumed | 12.962 | .000 | -1.592 | 404 | .112 | -20.6030 | 12.9410 | -46.0431 | 4.8372 |
|  | Equal variances not assumed |  |  | -1.386 | 189.250 | .167 | -20.6030 | 14.8598 | -49.9151 | 8.7092 |

| **Independent Samples Effect Sizes** | | | | | |
| --- | --- | --- | --- | --- | --- |
|  | | Standardizer^a^ | Point Estimate | 95% Confidence Interval | |
|  |  |  |  | Lower | Upper |
| VS | Cohen's d | 4.725 | -.287 | -.495 | -.078 |
|  | Hedges' correction | 4.734 | -.286 | -.494 | -.078 |
|  | Glass's delta | 5.370 | -.252 | -.461 | -.042 |
| SS | Cohen's d | 3.218 | -.174 | -.381 | .034 |
|  | Hedges' correction | 3.224 | -.173 | -.380 | .034 |
|  | Glass's delta | 3.339 | -.167 | -.375 | .041 |
| Total | Cohen's d | 7.162 | -.267 | -.475 | -.059 |
|  | Hedges' correction | 7.176 | -.267 | -.474 | -.059 |
|  | Glass's delta | 7.963 | -.240 | -.449 | -.031 |
| Delta Total | Cohen's d | 8.254 | -.046 | -.253 | .161 |
|  | Hedges' correction | 8.270 | -.046 | -.253 | .161 |
|  | Glass's delta | 8.207 | -.046 | -.253 | .161 |
| Post IPSS Q8 | Cohen's d | 1.314 | .016 | -.192 | .223 |
|  | Hedges' correction | 1.316 | .016 | -.191 | .223 |
|  | Glass's delta | 1.335 | .015 | -.192 | .223 |
| delta IPSS Q8 | Cohen's d | 1.537 | -.089 | -.296 | .119 |
|  | Hedges' correction | 1.540 | -.089 | -.295 | .118 |
|  | Glass's delta | 1.682 | -.081 | -.288 | .127 |
| Qmax | Cohen's d | 8.366 | .300 | .091 | .508 |
|  | Hedges' correction | 8.381 | .299 | .091 | .507 |
|  | Glass's delta | 7.508 | .334 | .122 | .544 |
| Delta Qmax | Cohen's d | 7.990005724189974 | -.117 | -.324 | .091 |
|  | Hedges' correction | 8.004877000226449 | -.117 | -.324 | .091 |
|  | Glass's delta | 7.937522396241836 | -.118 | -.325 | .090 |
| PVR' | Cohen's d | 24.932 | -.455 | -.664 | -.245 |
|  | Hedges' correction | 24.978 | -.454 | -.663 | -.244 |
|  | Glass's delta | 24.563 | -.462 | -.675 | -.246 |
| Delta PVR | Cohen's d | 122.3807 | -.168 | -.376 | .039 |
|  | Hedges' correction | 122.6085 | -.168 | -.375 | .039 |
|  | Glass's delta | 155.7833 | -.132 | -.340 | .076 |
| a. The denominator used in estimating the effect sizes.  Cohen's d uses the pooled standard deviation.  Hedges' correction uses the pooled standard deviation, plus a correction factor.  Glass's delta uses the sample standard deviation of the control group. | | | | | |
